# Future Environmental Performance of Lithium from Brines Shaped by Energy and Reagent Decarbonization and Technology Optimization

**DOI:** 10.1021/acs.est.6c09037

**Published:** 2026-09-10

**Authors:** Robert Istrate, Vanessa Schenker, Antoine Beylot, Victoire Collignon, Stephan Pfister, Bernhard Steubing

**Affiliations:** † Institute of Environmental Sciences (CML), 84813 Leiden University, Leiden 2333 CC, The Netherlands; ‡ Chair of Ecological Systems Design, Institute of Environmental Engineering, 27219 ETH Zurich, Zurich 8093, Switzerland; § 52810 BRGM, Orléans F-45060, France

**Keywords:** critical raw materials, lithium-ion batteries, prospective life cycle assessment, climate change, water scarcity, scenario analysis

## Abstract

The rapid expansion of battery-grade lithium carbonate (Li_2_CO_3_) production from brines raises increasing concerns about future environmental impacts, which must be assessed in alignment with project commissioning timelines. Here, we assess the life cycle climate change and water scarcity impacts for 24 operating and announced brine-based Li_2_CO_3_ projects, accounting for future technology optimization and economy-wide decarbonization across the energy and chemical reagent sectors. These developments could reduce climate change impacts of individual projects by 26–89% by 2035. Substantial project-level mitigation does not necessarily translate into lower impact intensity in the Li_2_CO_3_ supply mix of these projects, which depends on the success of planned production capacity. Technology optimization and economy-wide decarbonization can only offset the higher impacts from announced projects, while achieving meaningful cuts requires a transition to electrified heating. This shift, combined with more resource-intensive direct lithium extraction (DLE) technologies, may exacerbate water scarcity impacts beyond production sites. Building a sustainable lithium supply chain demands early mitigation to counterbalance higher impacts of upcoming projects, heat decarbonization as an immediate rather than long-term goal, accelerated decarbonization of soda ash and lime industries, and a supply chain-wide approach to water management.

## Graphical abstract

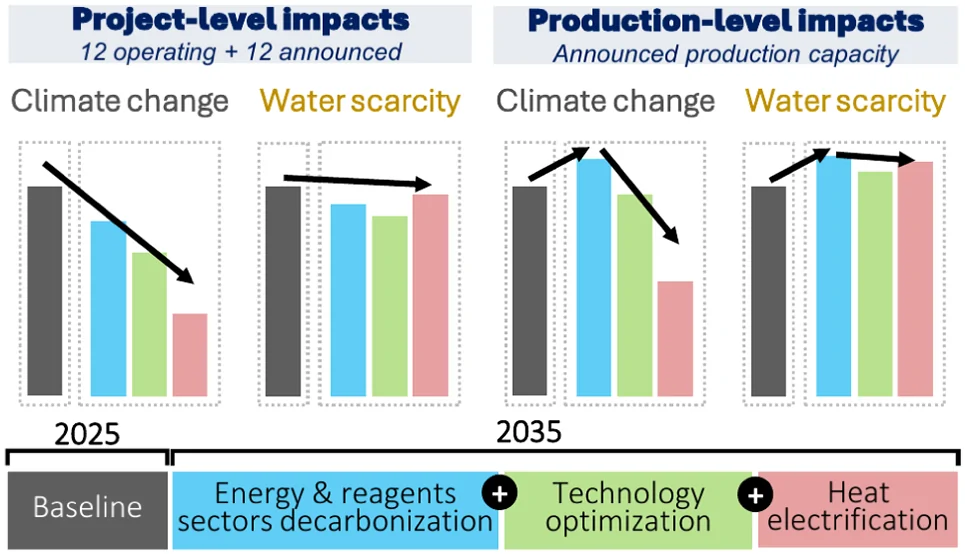

## 1 Introduction

Lithium is the raw material with the fastest-growing demand due to its essential role in lithium-ion batteries (LIBs) and the rapid adoption of electric vehicles (EVs).^1,2^ Meeting the increasing demand for battery-grade lithium carbonate (Li_2_CO_3_) and lithium hydroxide (LiOH) will require substantial expansion of mining and refining capacity,^1,3^ much of which is expected to come from lithium-bearing brines that account for more than 70% of global lithium resources.^4^ Life cycle assessment (LCA) studies indicate that brine-based lithium supply chains involve substantial greenhouse gas (GHG) emissions, due to energy-intensive processing and the extensive use of chemical reagents during lithium purification, and require intensive freshwater use, often in water-scarce regions such as the Atacama Desert.^5–9^ Emerging direct lithium extraction (DLE) technologies, designed to enhance access to lower-quality brines and improve lithium recovery efficiencies, may further exacerbate environmental impacts through higher energy, reagent, and water requirements.^10–12^

Currently, the Salar de Atacama in Chile, which hosts high-quality brines and has been identified as having relatively favorable environmental performance in existing LCA studies,^5,7^ accounts for over 60% of global brine-based lithium supply.^13^ In the near term, diversification toward Argentinean salt flats and Chinese salt lakes is expected, with resulting environmental impacts dependent on brine chemistry, technology choice, process configuration, and local conditions of new projects. Existing LCAs of selected operating and announced projects offer a good understanding of main impact drivers, including influence of lithium concentration and impurity levels (e.g., magnesium, calcium, and boron) on energy and reagents use;^5,7,8,14^ high influence of local context such as electricity supply, process heat fuel, and freshwater availability;^15^ substantial contribution of reagent production to overall impacts;^10,16^ and potentially higher impacts of DLE technologies such as solvent extraction, adsorption, nanofiltration, and membrane electrolysis.^10,12^

However, as many lithium projects remain in early development and large-scale expansion of both operating and announced operations is anticipated over the next decade,^17^ assessments based on current conditions may misrepresent future environmental performance at scale. Moreover, DLE technologies are still emerging,^18^ and their evaluation should account for the potential technology optimization. While some studies have explored low-carbon electricity and energy efficiency measures, they typically focus on existing mainstream projects (e.g., Salar de Atacama)^8,14,19,20^ or rely on average impact factors that do not capture project-specific characteristics such as brine chemistry or technology choice.^21^ As a result, the effects of future technological progress and broader economy-wide decarbonization have been largely overlooked; the latter includes systemic changes such as regional energy sector decarbonization aligned with climate policies or transformations in reagent production consistent with industry roadmaps. Moreover, environmental impacts will not be determined only by the performance of individual projects but also by how production is distributed across existing and new projects with heterogeneous characteristics. While existing LCAs suggest that announced projects may entail higher impacts than current operations, how these potentially higher impacts interact with project-level mitigation efforts and deployment dynamics to shape the overall environmental performance of the industry remains underexplored.

To address this knowledge gap, we assess the climate change and water scarcity impacts for 24 operating and announced projects producing battery-grade Li_2_CO_3_ from brines for 2025 and 2035. Using prospective LCA, we combine site-specific life-cycle inventories (LCIs), representing production technologies under both baseline and optimized conditions, with economy-wide decarbonization scenarios that reflect future changes in the energy sector, reagent production, and other industrial sectors. We further link project-level impacts with production timelines to evaluate the combined effects of planned capacity expansion and mitigation measures on the overall environmental performance. Our study contributes to advancing the understanding of the environmental implications of brine-based lithium supply under future conditions aligned with project commissioning.

## 2 Materials and Methods

Prospective LCA models the environmental performance of product systems over time by incorporating projected changes in technologies and markets based on scenarios of different plausible futures.^22^ This section presents the application of prospective LCA and scenario analysis in this study, following the four LCA phases according to the ISO 14040 standard.^23^

### 2.1 Goal and Scope

We conducted a prospective attributional LCA of climate change and water scarcity impacts using 1 kilogram of battery-grade Li_2_CO_3_ at the factory gate as a functional unit. Using cradle-to-gate system boundaries, we accounted for all inputs (electricity, heat, reagents, etc.) and outputs (emissions, waste streams, etc.) associated with brine extraction and concentration, brine purification, conversion to technical-grade Li_2_CO_3_, and purification to battery-grade Li_2_CO_3_. We analyzed 12 operating and 12 announced projects most likely to be in operation by 2035, spanning Chile, Argentina, Bolivia, China, USA, and Germany (Table S1). Operating projects are those reporting production as of April 2026, while announced projects are those still under development but with company-reported projected production by 2035. The selection of relevant projects was based on the S&P IQ Capital Pro database,^13^ with further details in Section 2.2.3 and Section 1 of the Supporting Information.

We assessed the environmental performance of the 24 projects for the reference year 2025 and three scenarios for 2035, considering the following changes (Table 1): (i) economy-wide decarbonization (hereinafter the background system) of energy, transportation, heavy industry, and reagent production sectors (*2035 Background*); (ii) optimization of Li_2_CO_3_ production technologies (*2035 +Optimized*); and (iii) electrification of low-temperature heat using either heat pumps or industrial electric boilers (*2035 +ElecHeat*). Future transformations in the energy, transportation, and heavy industry sectors are primarily based on projections from the Integrated Assessment Model (IAM) REMIND v2.1 ^24^ under a *middle-of-the-road* socioeconomic pathway (SSP2) and full implementation of the Nationally Determined Contributions (NDCs) under the Paris Agreement (see Section 2.2.2).^25^ We focus only on the REMIND SSP2-NDC scenario for simplicity, since previous research showed that short-term (2035) differences in impacts between IAMs and scenarios are relatively small.^26^ Future changes in reagent production were modeled to reflect industry decarbonization roadmaps and best available techniques (BAT). The technology optimization scenario focuses primarily on ambitious improvements in lithium recovery efficiency and reductions in energy and reagent consumption, and is therefore strongly oriented toward mitigating climate change impacts.

**Table 1:** Scenarios to Assess Future Project- and Production-Level Environmental Performance of Battery-Grade Li_2_CO_3_ Production from Brines

Scenario level	Scenario name	Scenario description
Project	2025	• Background system reflects current conditions (represented by reference year 2025)
		• Projects are modeled based on brine chemistry, process configuration, and technology performance as derived from technical reports and literature as of 2025
		• Natural gas is the primary fuel for process heating
	2035 Background	• Energy, transportation, and heavy industry sectors follow projections of technology performance, emission factors, and markets structure from the REMIND SSP2-NDC scenario and countries’ national energy plans. Reagent production follows trends consistent with industry decarbonization roadmaps and BAT performance levels
		• No progress in Li_2_CO_3_ production technologies or heat decarbonization
	2035 +Optimized	• Background system follows the same trajectory as in the *2035 Background* scenario
		• Li_2_CO_3_ production technologies are optimized to maximize lithium recovery efficiency and reduce energy and reagent consumption
		• No progress in heat decarbonization
	2035 +ElecHeat	• Background system and technology optimization follow the same trajectory as in the *2035 +Optimized* scenario
		• Low-temperature processes shift to either industrial heat pumps or electric boilers. Product drying, however, continues using natural gas or diesel, as electrifying high-temperature processes remains techno-economically challenging
Production	Operating	• Only projects operational as of April 2026 continue operation by 2035. It includes projects at the stages of “operating”, “limited production”, “preproduction”, “expansion”, and “satellite”
	Operating+ Announced	• All projects, regardless their current development stage, become operational by 2035 according to the production timeline projected by companies

To evaluate how planned capacity expansion and mitigation measures may jointly influence environmental impacts, we combine project-level impacts with production timelines under two scenarios: (i) a baseline including only operating projects and their expansion (*Operating*) and (ii) an extended case that additionally incorporates announced projects (*Operating+Announced*) (section 2.2.3).

### 2.2 Inventory Analysis

#### 2.2.1 Life-Cycle Inventory Modeling

The 24 projects cover three production technologies: evaporitic (e.g., Salar de Atacama), DLE (e.g., Salar del Hombre Muerto), and Li_2_CO_3_ production from geothermal brines (Upper Rhine Graben). In conventional evaporitic technology, brine is pumped to the surface and stored in ponds, where solar evaporation and mineral precipitation increase lithium concentration.^27^ Reagents such as quicklime may be added to the ponds to further remove impurities. The enriched brine undergoes multiple purification steps using chemical reagents to remove impurities, such as boron (precipitation with organic solvent) or magnesium and calcium (using soda ash). Technical-grade Li_2_CO_3_ is precipitated from purified brine using soda ash, while battery-grade Li_2_CO_3_ is obtained by dissolving the technical-grade material in water and reheating it to precipitate higher-purity Li_2_CO_3_.^10^ Some evaporitic operations also employ ion-exchange technology to remove impurities during brine processing (e.g., Salar de Olaroz). DLE technologies generally use physicochemical methods to recover lithium from low-grade brine. These include sorbents with high affinity for lithium cations, membranes for selective lithium recovery, nanofiltration, or electrochemical reactions.^28^ Typically, DLE technologies include brine pre-treatment (ion exchanger, precipitation, solvent extraction, or acidification) and final product post-processing (ion exchangers, precipitation, and volume reduction).^10^ Finally, geothermal brines are extracted from underground primarily for use in a geothermal power plant. The waste stream of the geothermal power plant could be sent to a DLE unit to extract lithium, after which the brine is reinjected into the reservoirs. Following the DLE process, additional purification steps are performed before precipitation of Li_2_CO_3_.^29^

LCIs for each project were obtained from the parametrized model developed by Schenker and Pfister.^10^ This model generates project-specific LCIs based on mass and energy balances, incorporating key technical and environmental parameters retrieved from company reports, patents, and the literature. These parameters include, among others, technology choice, process configuration, brine chemistry, recovery efficiencies, efficiency of chemical reactions, annual temperature, and process water recycling. For example, reagent consumption is estimated within the model based on stoichiometry while accounting for incomplete reactions and internal recycling rates, while heat demand is quantified based on thermodynamics, considering heat losses. A detailed description of the parametrized LCI model is provided in previous studies.^5,10,30^ In the current work, LCIs for 21 projects were directly adopted from Schenker and Pfister.^10^ For the remaining three projects (East Taijinair, Lakkor Tso Salt Lake, and Zhabuye), LCIs were developed using the same modeling framework (see Table S4 for input parameters). Moreover, all LCIs were revisited to implement project-specific electricity and heat supply sources, distinguishing between projects connected to the electricity grid and those relying on on-site generation (e.g., natural gas engines or diesel generators; Table S5).

Some projects may involve multifunctionality situations due to additional co-products alongside the main product. Brine concentration in evaporation ponds can yield potash as a co-product in addition to the enriched brine.^6^ However, data on potash co-production are largely lacking, with Salar de Atacama currently being the only site reporting such information. Thus, in this work, we allocate all environmental burdens from the evaporation ponds to the enriched brine. The geothermal brine used after the geothermal plant is treated as a waste stream, with the environmental burdens associated with extracting and reinjecting the brine fully allocated to the geothermal plant (see ref 30 for further details).

The impacts of the background system were quantified using the LCI database ecoinvent v3.10.1 (cutoff system model)^31^ combined with the open-source tool *premise* v2.2.9.^32^ *premise* updates the ecoinvent database by modifying key LCIs within the electricity, transport, biomass, fuels, heat, steel, and cement sectors, reflecting technological performance, emission factors, and market structure based on the REMIND SSP2-NDC scenario.^32^ We generated an adapted background LCI database for the reference year 2025, which served as the baseline for this study.

#### 2.2.2 Project-Level Scenario Modeling

##### 2.2.2.1 2035 Background

We used *premise* to generate a background LCI database for the year 2035 aligned with the REMIND SSP2-NDC scenario. However, an important limitation of using IAM scenarios is their high level of regional aggregation. REMIND includes China and the USA as distinct regions but groups Latin American countries together, making it difficult to account for future changes in the electricity mix in key lithium-producing countries like Argentina and Chile. To overcome this, we used country-specific projections for the electricity mix of Chile, Argentina, and Bolivia using each country’s national energy plans aligned with their NDC targets for 2035, combined with historical generation data for 2025 from the Ember Electricity Data Explorer.^33^ Although these scenarios were designed to reflect NDC targets as closely as possible, we acknowledge that inconsistencies may exist between these scenarios and those from REMIND.^34^ The electricity mix of each country in 2025 and 2035 is discussed in Section 3.1 and Table S6 in the Supporting Information.

Moreover, IAMs do not capture future changes in the production of key reagents. To address this, we integrated future changes in the production of quicklime, soda ash, sodium hydroxide, and hydrochloric acid based on industry decarbonization roadmaps and BAT performance levels (see Section 3.2 in the Supporting Information). We modeled quicklime production via the most-efficient vertical parallel flow regenerative kiln (PFRK),^35^ with limestone and process heat consumption reaching BAT levels by 2035.^36^ The fuel mix for the lime kiln will gradually shift from coal and oil to higher shares of natural gas and biomass,^37,38^ while a moderate share of the kilns (15%) are assumed to integrate carbon capture and storage (CCS) by 2035. Both natural and synthetic soda ash are considered, representing 33% and 67% of current global production, respectively.^39^ By 2035, the share of natural soda ash increases slightly to 38%. At present, about 60–75% of global soda ash production relies on coal, either for trona ore calcination (natural route) or for steam production in the Solvay process (synthetic route).^40,41^ In line with company decarbonization plans, coal use declines to a residual 5% by 2035, with natural gas and biomass providing 75% and 20% of process heat, respectively. Optimization of the Solvay process will further reduce raw materials and energy consumption, achieving BAT levels by 2035,^42^ while CCS integration applies to 15% of global production. About 99% of global sodium hydroxide is co-produced with chlorine through the chlor-alkali process, which involves the electrolysis of sodium chloride brine; chlorine also serves as a feedstock for hydrochloric acid production.^43^ We assumed that the more efficient membrane cell technology will fully replace diaphragm and mercury cell technologies in the chlor-alkali industry by 2035.^44^

##### 2.2.2.2 2035 +Optimized

This scenario aims to assess the effects of ambitious improvements in lithium recovery efficiency and reductions in energy and reagent consumption driven by technology optimization, alongside broader transformations in the background system. On the basis of the list of key parameters identified in Schenker and Pfister,^10^ we defined the following rules to implement technological improvements. First, overall lithium recovery rates are assumed to increase by 20% relative to the baseline rates for evaporitic technologies (baseline varying from 35% to 61%, depending on the project)^10^ and from the baseline of 77% (based on pilot-scale testing results from Salar de Tolillar)^45^ to 95% for DLE technologies. The 95% recovery rate for DLE technologies is obtained from Vera et al.,^28^ who compiled recovery rates from laboratory- and pilot-scale studies reported in the literature. This recovery rate is considered highly ambitious and is used as a best-case scenario to explore the potential environmental improvements that can be achieved. Importantly, higher recovery rates reduce the mass flows throughout the processes, thereby affecting the demand for energy, reagents, and freshwater. Second, process efficiencies are increased to a maximum. Third, chemical reactions are optimized, reducing the required surplus of reagents to <1%. Fourth, it is assumed that effluents from processes such as reverse osmosis, adsorption, or evaporator units achieve higher lithium concentrations, further reducing the required mass flows. Finally, electricity consumption in equipment (e.g., in centrifuges or DLE technologies) decreases to 90% of the baseline values. The values of the key parameters and an overview of the energy and reagents demand and water flows under the baseline and optimized conditions for each project can be found in Tables S7 and S8, respectively.

##### 2.2.2.3 2035 +ElecHeat

This scenario assumes that processes requiring low temperatures, such as the precipitation step (80°C), will transition from natural gas to electrified heat. We consider two technological options: heat pumps and electric boilers. Heat pumps are widely used for industrial applications below 100 °C and represent a readily available option.^46^ We modeled this shift using the ecoinvent LCI for heat pumps, relinking electricity inputs to the project-specific electricity supply. We further test the results by considering industrial electric boilers instead of heat pumps. Electric boilers achieve efficiencies of up to 99%,^47^ but they are still less efficient than heat pumps, thereby representing a more conservative assumption. In all cases, product drying was assumed to continue relying on fossil fuels (natural gas or diesel), as decarbonizing high-temperature processes remains techno-economically challenging.^48^

#### 2.2.3 Production-Level Scenario Modeling

The 24 projects were selected from the S&P IQ Capital Pro database,^13^ from where we also extracted project-specific information on development stage and expected production timelines through 2035 (data retrieval approach detailed in Section 1 of the Supporting Information). The selected projects span a range of development stages, including “operational”, with “planned expansions”, “under construction” or with “construction planned”, or projects that have completed feasibility studies (Table S1). These development stages were used to assign projects to the *Operating* and *Operating+Announced* scenarios as defined in Table 1 (with further details in Table S9). Figure 1 shows the production of battery-grade Li_2_CO_3_ from the 24 projects under both scenarios, disaggregated by country and technology, with the technology breakdown based on the classification assigned to each project (Table S1).

**Figure 1:**
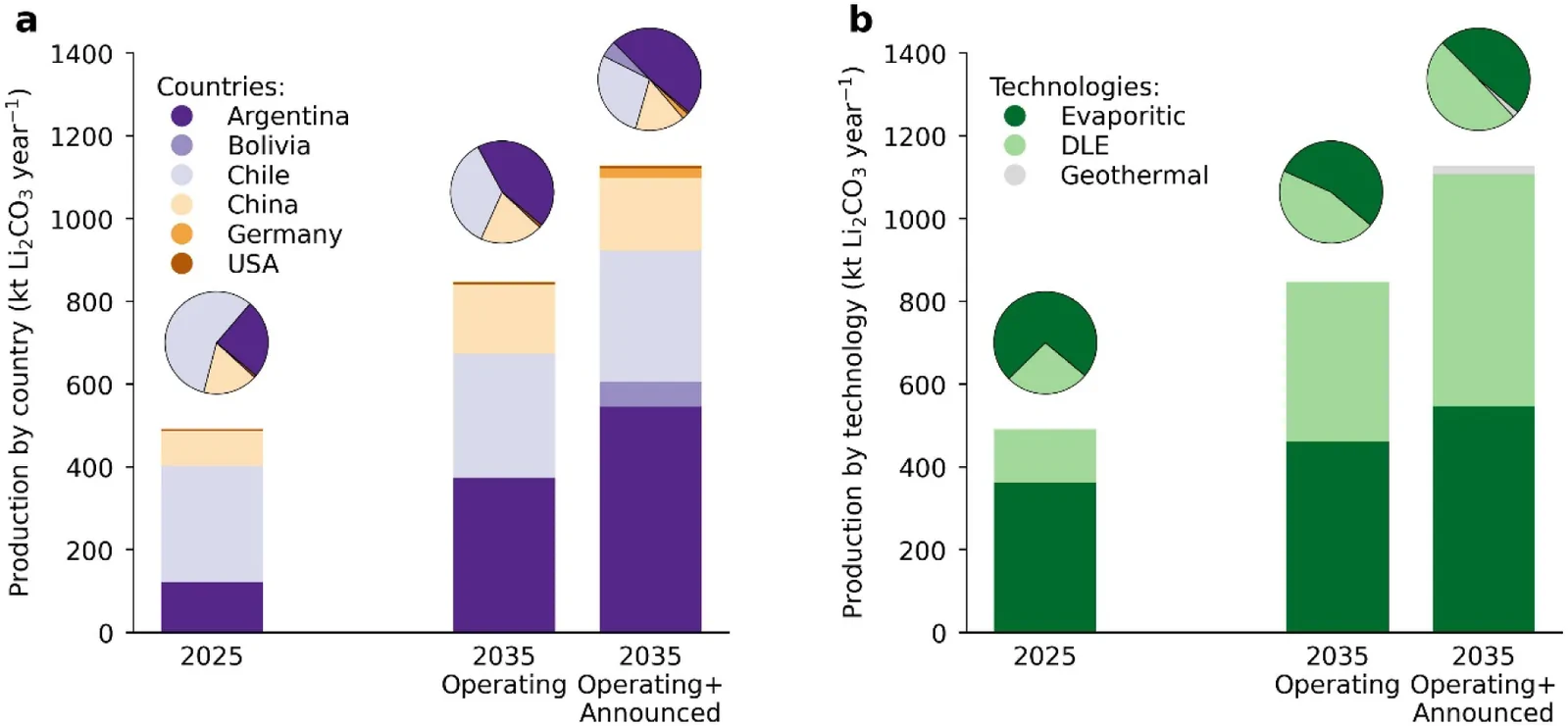
Production-level scenarios for battery-grade Li_2_CO_3_ production from brine based on 24 operating and announced projects. Annual production is disaggregated by country (a) and technology (b), with pie charts indicating relative contributions. “2035 Operating” represents production in 2035 considering production and expansions from the 12 projects classified as operational as of April 2026. “2035 Operating + Announced” includes all 24 projects. DLE: direct lithium extraction.

The *Operating* scenario includes only the 12 projects classified as “operational” as of April 2026, along with their planned expansion, resulting in production rising from 491 to 848 kt Li_2_CO_3_ year^–1^ between 2025 and 2035. Chile (represented only by Salar de Atacama) accounts for 57% of this annual production in 2025, followed by Argentina with 25% (six projects), China with 17% (four projects), and the USA with <1% (one project). Chile’s share declines to 35% by 2035, while Argentinean and Chinese projects expand production to reach 44% and 20% share, respectively; the USA remains at <1% (Figure 1a). Five of the operational projects employ conventional evaporitic technologies, accounting for 74% of production in 2025, while the remaining seven use DLE technologies and contribute 26%. By 2035, evaporitic technologies still dominate in this scenario, although their share decreases to 54% while DLE increases to 46% (Figure 1b). It should be noted that these shares are relative to the total production of the 24 brine-based projects included in our study. They would be lower if global production were considered, including production from the hard-rock route.

In the *Operating+Announced* scenario, inclusion of announced projects results in a 33% increase in production by 2035, reaching 1,128 kt Li_2_CO_3_ year^–1^. Argentina surpasses Chile in this scenario, reaching 48% production share by 2035 while Chile’s share drops to 28%. China’s share remains relatively stable in this scenario. However, the dataset used in this study may not fully cover all ongoing Chinese projects.^49^ Projects in other countries, including the USA, Bolivia, and Germany, would hold less than 5% of production share each by 2035. The share of production from evaporitic technology decreases to 49% by 2035, while DLE’s contribution grows to about 49%.

### 2.3 Impact Assessment

We calculated climate change impacts using 100-year global warming potentials (GWPs) from the Intergovernmental Panel on Climate Change (IPCC) Sixth Assessment Report (AR6),^50^ with a GWP of −1 assigned to CO_2_ uptake from air and +1 to biogenic CO_2_ emissions to ensure carbon balance. Water scarcity impacts were quantified with the Available WAter REmaining (AWARE) method, which quantifies the potential of freshwater deprivation at the basin, country, and global levels by comparing human and environmental water demand to water availability.^51^ Accordingly, water scarcity impacts are directly proportional to freshwater consumption (i.e., water lost to the ecosystem via evaporation or incorporation in products, which depends on the amount of freshwater use quantified in the LCIs) and inversely proportional to the available water remaining in a region. We used basin-level characterization factors (nonirrigation) to quantify the impacts of foreground processes, thus accounting for the specific water scarcity at the lithium project site (Table S10). In contrast, we quantified impacts linked to background processes by using the global average characterization factor (unspecified). According to the UNEP-SETAC guidelines, only freshwater should be assessed when using AWARE,^51^ and there is a clear consensus that the high mineral content of brine makes it unfit for human consumption. Therefore, in this study, brine water evaporation is not considered when computing water scarcity impacts (see Section 4 in the Supporting Information for additional discussion).^5–7^ The implementation of the LCIs and LCA calculations was performed with *Brightway2.5* v2.4.6, a Python-based open-source LCA software.^52^

## 3 Results and Discussion

### 3.1 Project-Level Climate Change Impacts

Using 2025 as the reference year, the climate change impacts of the 24 projects range from 4.3 to 74 kg CO_2_-eq kg^–1^ Li_2_CO_3_ (Figure 2). These values are consistently higher than previous estimates (2.4–51 kg CO_2_-eq)^10^ primarily due to the consideration of project-specific electricity and heat sources, updated LCIs for reagent production and electricity mixes, and a different background LCI database in this work. Nevertheless, the trends identified by Schenker and Pfister^10^ persist: projects employing DLE technologies exhibit higher impacts than those based on conventional evaporitic technologies, primarily due to higher heat and reagent requirements for processing lower-quality brines.

**Figure 2:**
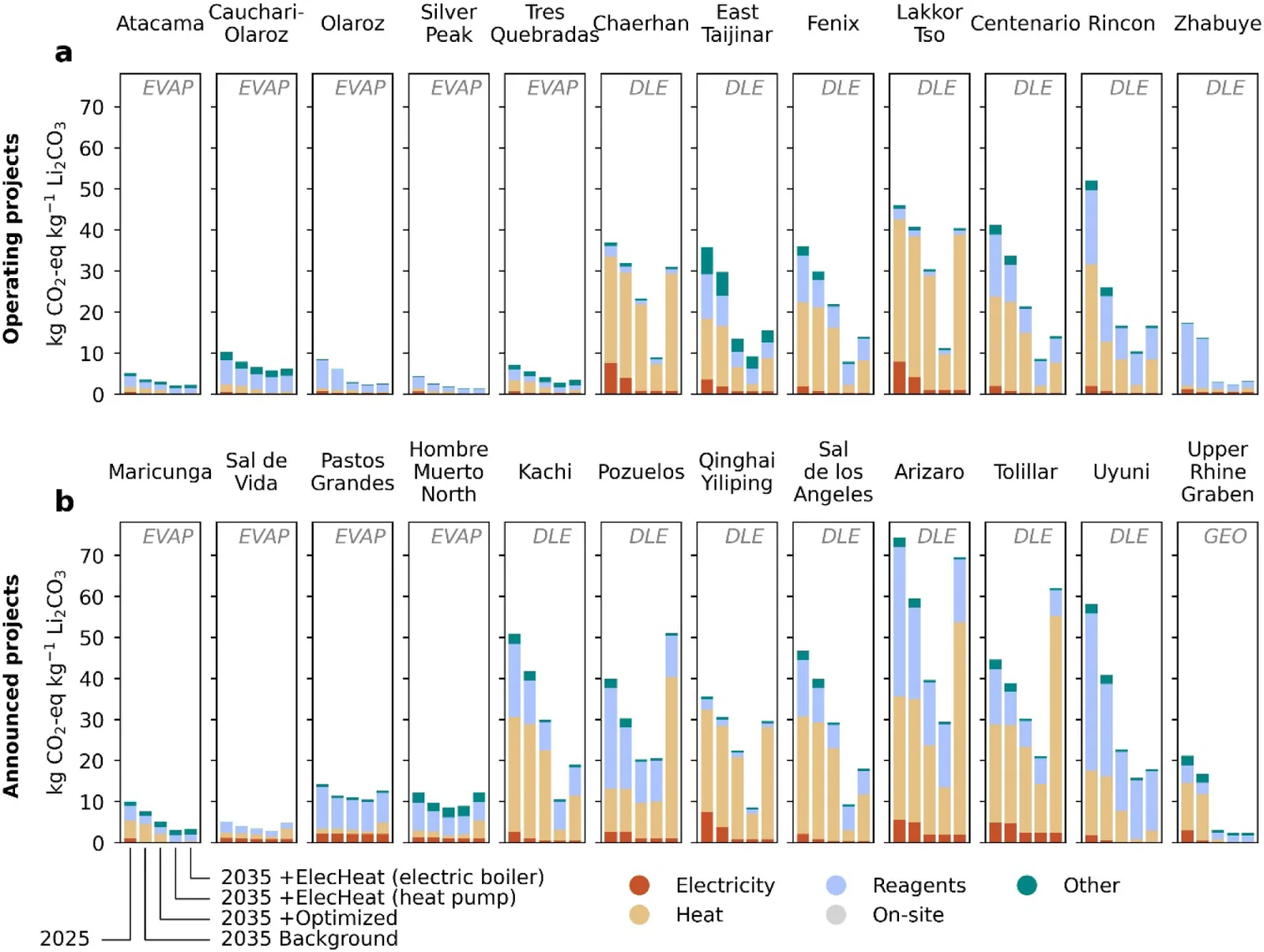
Life cycle climate change impacts of operating (a) and announced (b) battery-grade Li_2_CO_3_ production projects in 2025 and 2035. 2035 impacts are quantified considering three alternative scenarios: (i) only background system transformations (2035 Background); (ii) background system transformation plus Li_2_CO_3_ production technology optimization (2035 +Optimized); and (iii) previous changes plus a switch to electrified low-temperature heat using either heat pumps or electric boilers (2035 +ElecHeat). EVAP: evaporitic; DLE: direct lithium extraction; GEO: geothermal.

The changes considered in our 2035 scenarios could mitigate 26–89% of the climate change impacts, resulting in 1.3–29 kg CO_2_-eq kg^–1^ Li_2_CO_3_. Future changes in the background system account for 12–50% of this reduction (*2035 Background* in Figure 2). Projects where reagents are the main hotspot (e.g., Silver Peak or Salar de Atacama) benefit most from background system transformations due to the decarbonization of reagent production (Figure S3). The life-cycle GHG emissions of sodium hydroxide, hydrochloric acid, and sulfuric acid production decrease by 43%, 31%, and 23%, respectively, primarily due to global electricity decarbonization (Figure S4). Moreover, process optimization, fuel switching, and CCS adoption lower the impacts of quicklime by 21% and soda ash by 42%. In contrast, projects where heat is the major hotspot (e.g., Lakkor Tso or Sal de los Angeles) see limited impact reductions as heat-related emissions remain largely unchanged. Changes in natural gas supply reduce impacts by only <1%, whereas emissions from gas or diesel combustion do not change. Electricity consumption remains a minor contributor to impacts as the expansion of renewable power generation over the next decade reduces the GHG emissions intensity of the national electricity mix by 86% in Chile, 49% in China, or 60% in Argentina (Figure S4). The mitigation potential of grid decarbonization is more impactful for Chinese projects, since electricity already plays a limited role in other countries by 2025.

Combining background system transformations with technology optimization more than doubles climate change impacts mitigation, achieving 22–85% reduction relative to the 2025 situation (*2035 +Optimized* in Figure 2). This scenario reduces electricity, heat, and reagent consumption through enhanced lithium recovery rates, process and energy efficiency, and optimized chemical reactions (Table S8). The wide range in mitigation potential primarily stems from disparities among technologies. Projects based on DLE or ion-exchange technologies (e.g., Zhabuye, 83% mitigation; Salar del Rincon, 68%; Salar de Olaroz, 66%) tend to benefit more from technology optimization than conventional evaporitic operations (e.g., Salar de Atacama, 42%; Salar de Pastos Grades, 22%). The high mitigation potential for DLE technologies stems primarily from the substantial increase in lithium recovery efficiency assumed in this scenario (from 77% to 95%).

The effectiveness of low-temperature heat electrification (*2035 +ElecHeat* in Figure 2) depends largely on both the electrification technology as well as the existing heat source and the GHG emissions intensity of the electricity supply used by each project. Replacing existing heating with heat pumps reduces climate change impacts across most projects, with the largest benefits observed for Argentinean and Chinese projects connected to the electricity grid and initially relying on natural gas for heat supply (Figure S5). In contrast, impacts increase for Salar del Hombre Muerto North due to its reliance on diesel-based electricity generation and for Pozuelos because its current heat supply is already relatively low-carbon (based on natural gas combined heat and power). For most projects in Chile, Argentina, and Bolivia, heat-related emissions become relatively minor after adopting heat pumps, leaving reagent-related emissions as the dominant sources of impacts in the future. However, when industrial electric boilers are implemented instead of heat pumps, climate change impacts can increase substantially, in some cases exceeding those observed under the *2035 +Optimized* scenario. This is particularly evident for off-grid projects relying on diesel- or natural-gas-based electricity generation, as well as for Chinese projects affected by a relatively carbon-intensive electricity mix by 2035.

### 3.2 Project-Level Water Scarcity Impacts

Water scarcity impacts in 2025 range from 0.7 to 43 m^3^_world_-eq kg^–1^ Li_2_CO_3_ (Figure 3). However, Salar del Rincon is an outlier, as it is the only project currently considering electric boilers, which associated electricity demand drives its comparatively higher water scarcity impacts. Excluding this project, the upper bound decreases to 34 m^3^_world_-eq kg^–1^ Li_2_CO_3_ (corresponding to Pozuelos), bringing our range in line with the 0.8–29 m^3^_world_-eq kg^–1^ Li_2_CO_3_ reported by Schenker and Pfister.^10^

**Figure 3:**
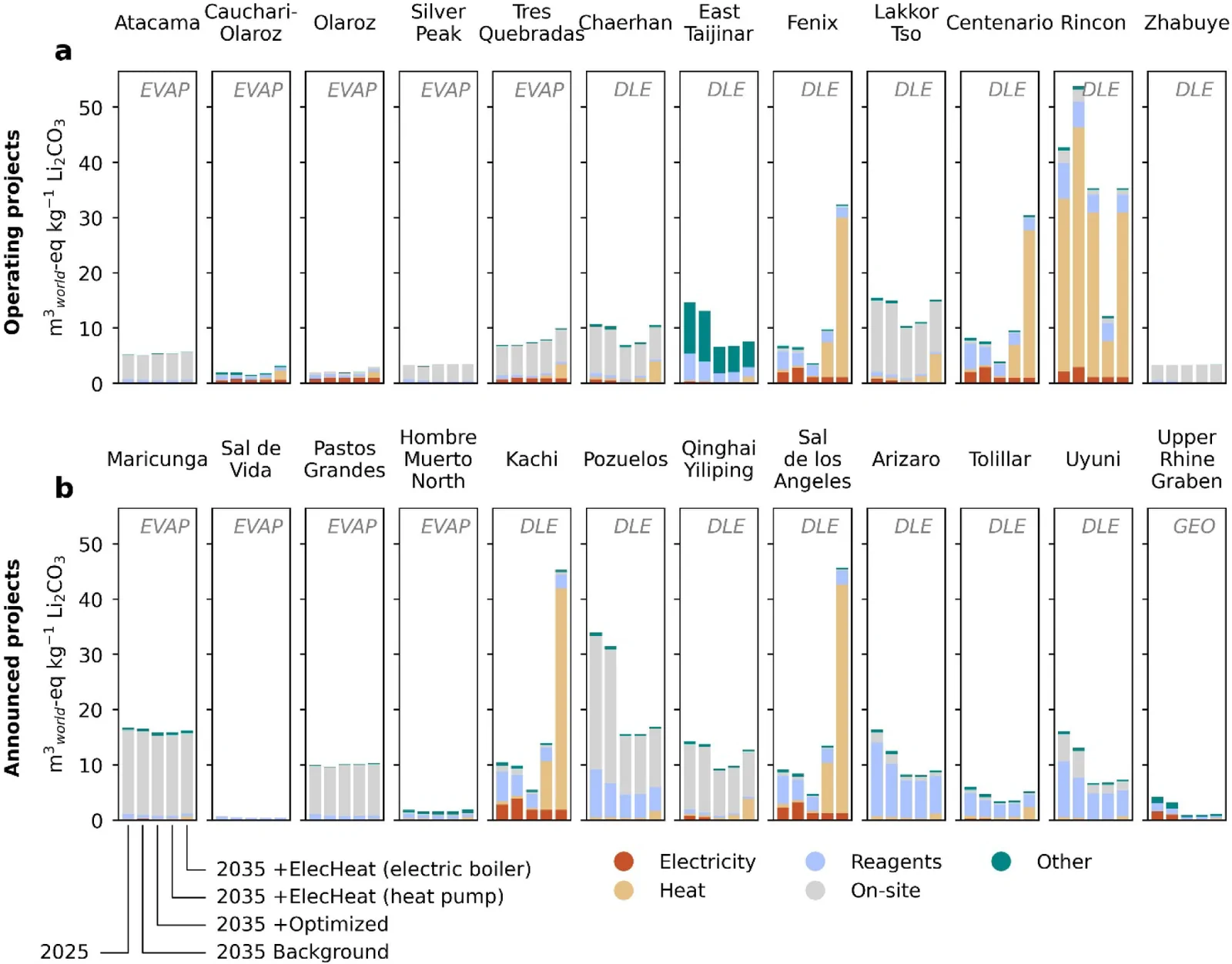
Life cycle water scarcity impacts of operating (a) and announced (b) battery-grade Li_2_CO_3_ production projects in 2025 and 2035. 2035 impacts are quantified considering three alternative scenarios: (i) only background system transformations (2035 Background); (ii) background system transformation plus Li_2_CO_3_ production technology optimization (2035 +Optimized); and (iii) previous changes plus a switch to electrified low-temperature heat using either heat pumps or electric boilers (2035 +ElecHeat). EVAP: evaporitic; DLE: direct lithium extraction; GEO: geothermal.

Water scarcity impacts do not show a consistent trend by 2035, ranging from a 78% decrease (Upper Rhine Graben) to 47% increase (Sal de los Angeles) when all future transformations are considered (*2035 +ElecHeat* scenario with heat pumps). The effect of background system transformations ranges from 25% reduction (Upper Rhine Graben) to 26% increase (Salar del Rincon) in impacts. Changes in the background system offer limited impact reduction for projects in areas with high water scarcity, such as Salar de Atacama (3% reduction), where extreme aridity results in a characterization factor of 94.7 m^3^_world_-eq m^–3^ and on-site freshwater consumption dominates impacts. Conversely, projects in regions with lower characterization factors, such as some Argentinean sites, are more influenced by background changes. As with climate change, the largest reductions occur for projects where reagents are major contributors to water scarcity impacts, such as Sal de Vida or Salar de Arizaro (24% reduction each; Figure S6). These reductions are primarily driven by improvements in water-use efficiency in soda ash production (reducing soda ash water scarcity impacts by 29%) and the phase-out of diaphragm technology in the chlor-alkali industry (reducing sodium hydroxide impacts by 63% and hydrochloric acid by 28%; Figure S4). A slight increase in electricity-related water scarcity impacts is observed for several Argentinean projects (e.g., Fenix, Salar de Centenario, and Salar del Rincon) due to a 38% increase in the water scarcity impacts of the Argentinean electricity mix between 2025 and 2035 (Figure S4). This increase is primarily due to the growing share of hydropower in the grid (from 19% in 2025 to 28% by 2035).

Technology optimization shows mixed effects on water scarcity impacts, depending on the technology applied, with changes ranging from 71% reductions to 13% increases relative to background system changes. Projects employing DLE technologies benefit most (e.g., Pozuelos, 50% reduction; Kachi, 44%) because both on-site freshwater consumption and reagent demand decrease with technology optimization (Figure S6). However, because of their relatively higher water use compared with conventional evaporitic technologies, they still exhibit higher impacts even under optimized conditions. Limited impact mitigation observed for certain projects (e.g., Salar de Atacama, 8% increase; Maricunga, 4% reduction) is largely attributed to evaporative losses during water treatment and purification and liquid waste storage, factors that remain largely unchanged. Notably, the technology-optimization scenario modeled in this work primarily targets overall efficiency gains to reduce climate change impacts, which in some cases may increase wastewater generation and, consequently, water scarcity impacts.

Switching to heat pumps increases water scarcity impacts for most projects relative to the scenario that combines background system changes with technology optimization, primarily due to the additional electricity consumption for heat pump operation. The increase is modest for most projects, remaining below 1% for seven projects and below 10% for 15 projects. The most pronounced increases are observed for a small number of grid-connected projects in Argentina (e.g., Sal de los Angeles, 184% increase; Fenix, 164%), where the national grid’s water scarcity impacts rise by 2035. For several projects (e.g., Tres Quebradas, Fenix, and Kachi), the added impacts from heat pumps largely offset the reductions achieved through other changes, resulting in higher overall water scarcity impacts compared to 2025. Shifting to electric boilers instead of heat pumps would considerably exacerbate these impacts due to higher electricity consumption. These findings highlight a key trade-off: while electrified low-carbon heat can significantly reduce climate change impacts, it may simultaneously exacerbate water scarcity impacts, particularly in regions with water-intensive electricity generation.

### 3.3 Production-Level Environmental Performance

The production-level climate change impacts of the 12 projects operating in 2025 (i.e., project-level impacts weighted by production volumes) are estimated at 14 kg CO_2_-eq kg Li_2_CO_3_^–1^ (Figure 4a-c), closely matching the 15 kg CO_2_-eq kg Li_2_CO_3_^–1^ reported by Schenker and Pfister.^10^ Across the combined project- and production-level scenarios for 2035, impacts range from 5.6 to 20 kg CO_2_-eq kg Li_2_CO_3_^–1^, reflecting that both the adoption of mitigation measures as well as the success of announced production capacity play a role in shaping the future climate change impacts of Li_2_CO_3_ supply.

**Figure 4:**
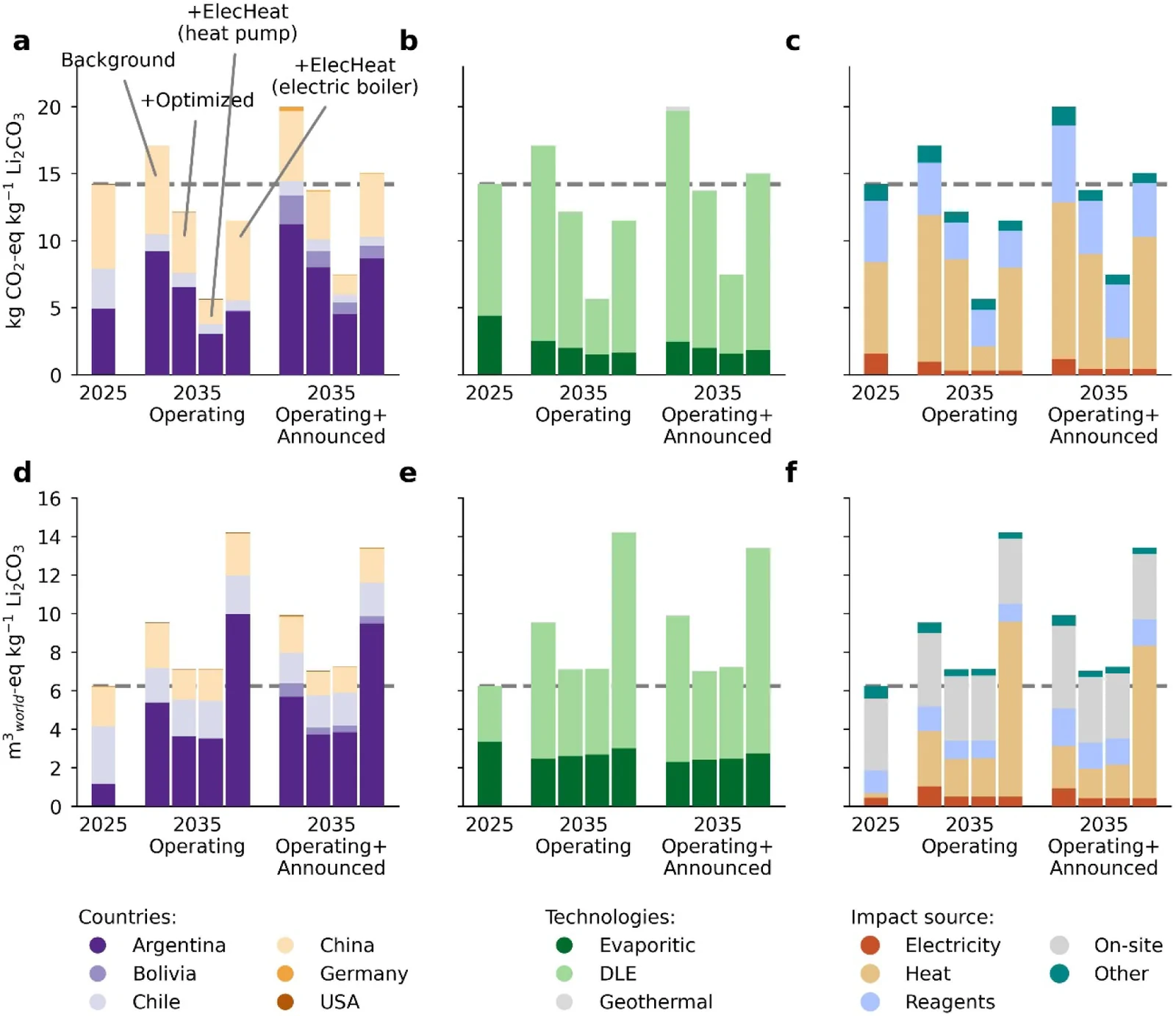
Production-level life cycle climate change (a–c) and water scarcity (d–f) impacts of battery-grade Li_2_CO_3_ production under alternative scenarios. Production-level impacts are calculated as project-level impacts weighted by production volumes across the 24 projects. Impacts are disaggregated by country of the project (a, d), technology (b, e), and impact source (c, f). “2035 Operating” represents production in 2035 considering production and expansions from the 12 projects classified as operational as of April 2026. “2035 Operating+Announced” includes all 24 projects. Moreover, 2035 impacts are quantified considering the three project-level scenarios (2035 Background, 2035 +Optimized, and 2035 +ElecHeat (using either heat pumps or electric boilers). DLE: direct lithium extraction.

Production-level climate change impacts increase in both the *Operating* and *Operating+Announced* scenarios when only background system transformations are considered (by 20% and 41% relative to 2025, respectively). This indicates that higher impacts associated with expanding existing projects or introducing announced projects processing lower-quality brines (primarily in Argentina) can offset reductions achieved through the decarbonization of energy or reagent supply (Figure 4a). Further technology optimization also appears insufficient for deep GHG emissions reductions by 2035, reducing impacts by 15% in the *Operating* scenario but only 3% when announced projects are included. Thus, the substantial project-level mitigation potential achieved through economy-wide decarbonization and technology optimization (Figure 2) does not necessarily translate into proportional reductions in the production-level impact intensity. Such reductions are only achieved with a transition to heat pumps, which delivers reductions of 47–60% relative to 2025. However, if heat electrification is implemented via electric boilers instead, production-level impacts decrease by 19% in the *Operating* scenario but increase by 6% in the *Operating+Announced* scenario.

The production-level water scarcity impacts of the 12 operating projects in 2025 are estimated at 6.3 m^3^_world_-eq kg Li_2_CO_3_^–1^ (Figure 4d-f). For the 2035 alternative scenarios, impacts range from 7 to 14 m^3^_world_-eq kg Li_2_CO_3_^–1^. Like climate change, production-level impacts increase when considering only transformations in the background system (by up to 59% in the *Operating+Announced* scenario). This increase is largely driven by production expansion in Argentina, where projects, despite having relatively low site-specific characterization factors, are more energy- and reagent-intensive. Thus, the water scarcity impacts increase is mainly driven by freshwater consumption from electricity generation and reagent production, whereas the impacts from on-site freshwater consumption remain nearly constant (Figure 4f). Technology optimization could help counterbalance this trend, maintaining 2035 impacts close to those of 2025 by reducing reagent consumption. Electrification of low-temperature heat with heat pumps shows similar impacts as the *2035 +Optimized* scenario, yet using electric boilers would increase impacts by 115–128% relative to 2025. These increases are driven by the additional freshwater consumption associated with electricity generation for the operation of electric boilers.

### 3.4 Impact Mitigation Opportunities and Challenges

Our prospective analysis indicates that the high climate change impacts of announced brine-based Li_2_CO_3_ projects identified in previous research (notably those based on DLE technologies)^10^ could be substantially reduced over the next decade. Although the effectiveness of decarbonization strategies varies according to the specific characteristics of each project (reflected in mitigation potentials ranging from 26% to 89%), several overarching decarbonization recommendations have emerged. However, our results also suggest a potential trade-off, as strategies aimed at reducing life-cycle GHG emissions may further exacerbate water scarcity impacts.

#### 3.4.1 Heat Decarbonization: An Immediate Imperative for Climate Goals with Water Scarcity Implications

Our findings suggest that electricity consumption contributes only a minor share of life-cycle GHG emissions for the assessed projects. For grid-connected projects, ongoing policies aimed at decarbonizing national electricity grids can substantially reduce these emissions (as represented here by REMIND’s SSP2-NDC scenario and countries’ energy plans). For off-grid projects relying on natural gas or diesel for on-site electricity generation, a shift to solar or wind power generation alone would result in limited reduction in overall GHG emissions. Instead, decarbonization of process heat emerges as a key lever for achieving meaningful GHG emissions reductions. The projects modeled in this work, based on technical reports, show high heat demand and large reliance on natural gas or diesel. While technology optimization can reduce heat requirements, achieving deep emissions cuts will require transitioning to low-carbon heating alternatives. This urgency contrasts with industry roadmaps, which often classify process heat decarbonization as a medium- to long-term goal^53^ due to the techno-economic challenges associated with high-temperature processes.^46^ In the context of lithium, these challenges are primarily discussed for the hard-rock route.^54^ However, multiple low-carbon options are already available to meet the low-temperature requirements in brine-based Li_2_CO_3_ production, including industrial heat pumps.^19^ Heat pumps are preferred over electric boilers because the latter’s lower efficiency can lead to increased GHG emissions depending on the carbon intensity of electricity supply. Moreover, additional electricity consumption for electrified heat can increase water scarcity impacts in countries with high shares of hydropower, such as Argentina. While these impacts occur at the hydropower generation site rather than the lithium site itself (therefore, different stakeholders), they represent an important trade-off that must be considered when evaluating the feasibility of heat decarbonization.

#### 3.4.2 Set Scope 3 Emissions Targets and Accelerate Reagent Decarbonisation

Low-carbon reagents are critical for decarbonizing the lithium industry. Some reagents, such as sodium hydroxide and hydrochloric acid, will benefit from the decarbonization of the power sector since their impacts are largely driven by electricity consumption.^55^ Others are harder to decarbonize due to large process- and heat-related CO_2_ emissions. Soda ash and quicklime production release direct CO_2_ emissions from limestone calcination and rely heavily on fossil-fuel heat.^35,40^ In this study, we assumed optimistic transformations in the production of these reagents by 2035, including an almost full shift away from coal, technology improvements aligned with best BAT performance, and partial CCS adoption. Figure 5 illustrates that alternative developments for these reagents could have large effects on the climate change mitigation potential achieved by 2035. For example, impacts for Salar de Uyuni range from a 23% decrease to an 18% increase depending on how soda ash and quicklime will be produced. These findings highlight the lithium industry’s strong dependence on future decarbonization efforts within the chemical reagents sector. However, despite their relevance, reagent-related emissions are often overlooked in the mining and metals industry decarbonization roadmaps, which typically focus on Scope 1 and 2 emissions while neglecting Scope 3.^53^ Moreover, while the European and US lime industries have published decarbonization roadmaps,^38,56–58^ similar efforts are lacking for lime production in lithium-producing countries and for soda ash, highlighting significant uncertainty regarding their future decarbonization pathways.

**Figure 5:**
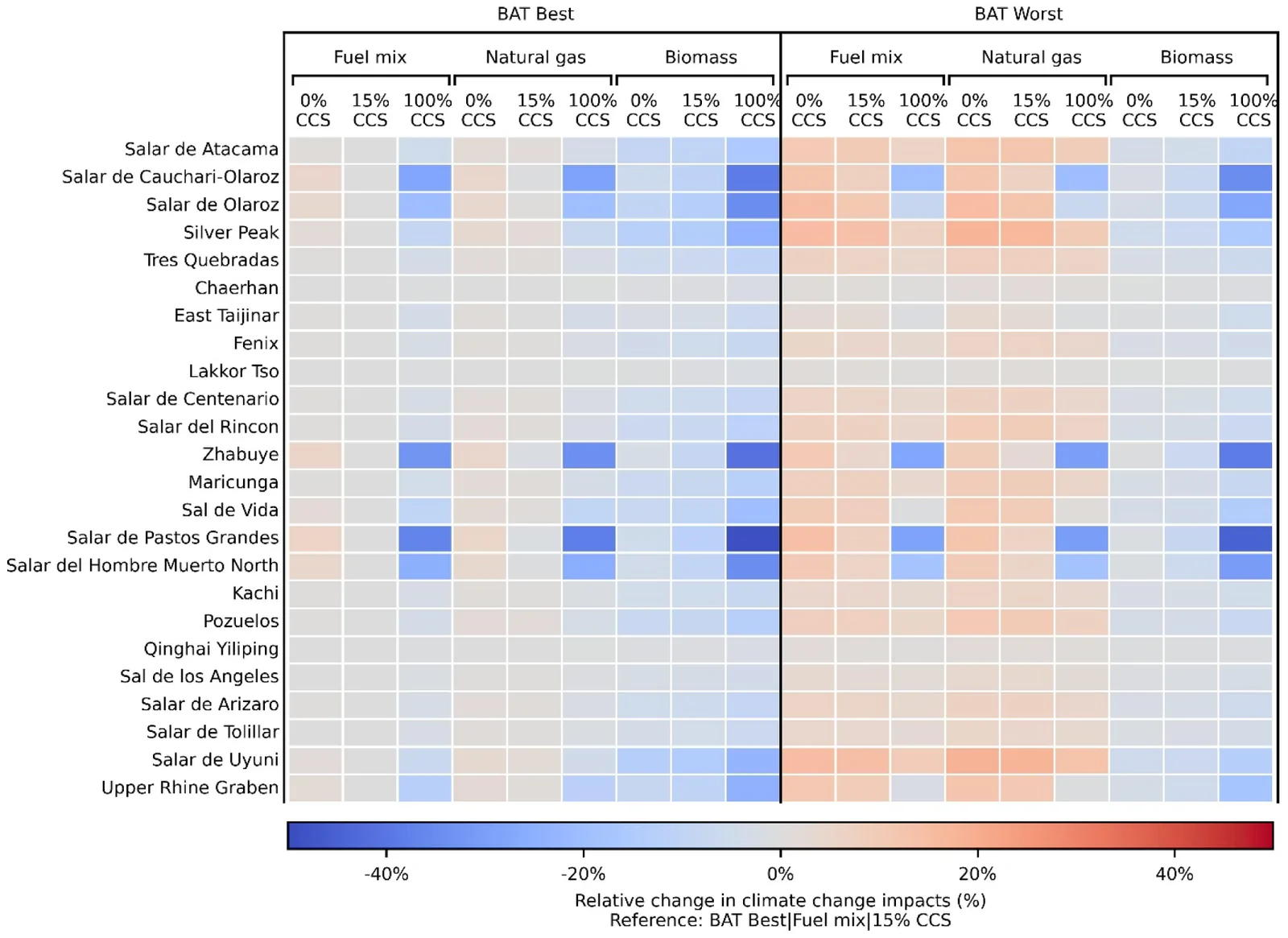
Sensitivity of project-level life-cycle climate-change impacts to variations in soda ash and quicklime production by 2035. The 2035 +Optimized scenario is used as reference, assuming production of soda ash and quicklime based on best BAT performance, fuel mix, and 15% CCS. The remaining columns represent 17 alternative combinations of performance level (best vs. worst BAT), fuel source (mix, 100% natural gas, and 100% biomass), and CCS adoption (0%, 15%, and 100%).

#### 3.4.3 Adopt a Supply Chain-Wide Perspective on Water Scarcity

Freshwater consumption linked to material production, including metals, has doubled between 1995 and 2021, calling for urgent water management measures to improve efficiency.^59^ For example, the Chilean mining sector, where water scarcity is a critical issue, has faced strong incentives to improve water efficiency, as evidenced by the substantial reduction in water demand by the copper mining industry over time.^60^ A recent study conducted at Salar de Atacama found a 19% reduction in blue water demand intensity between 2020 and 2023 due to an increased brine recovery efficiency.^61^ Extending the analysis to life-cycle water scarcity impacts across various Li_2_CO_3_ projects reveals key challenges. Targeting the optimization of freshwater use in DLE technologies at projects in arid regions of Chile, Argentina, and China offers the highest local mitigation potential. However, these projects are also more energy- and reagent-intensive, exacerbating water scarcity impacts beyond those at production sites. Moreover, trade-offs from climate change mitigation measures, such as switching to heat pumps, could further shift the impacts. Under such a scenario, water scarcity from electricity and reagent production may become as significant as on-site impacts, challenging the current focus predominantly on on-site freshwater consumption.^6,7,15^

#### 3.4.4 Implications for the Environmental Footprint of LIBs and EVs

If all announced production capacity considered in this study is achieved, the 24 projects would reach 1,128 kt Li_2_CO_3_ year^–1^, resulting in an increase in the annual climate change impacts from 6.9 Mt CO_2_-eq in 2025 to 23 Mt CO_2_-eq by 2035 without technology optimization and low-carbon heat. These impacts would remain modest when compared with the potential savings from replacing internal combustion engine vehicles with EVs, which could reach 1.8 Gt CO_2_-eq in 2035 under the IEA’s STEPS scenario.^62^ However, the future climate change impacts of Li_2_CO_3_ supply have important implications for LIB batteries, particularly under the EU Batteries Regulation, which intends to set carbon footprint thresholds for new batteries put on the EU market.^63^ Previous studies indicate that Li_2_CO_3_ contributes about 13% to the life cycle climate change impacts of LFP cells and 10% for NMC811 cells.^9,64^ Our results show that further technology optimization and low-carbon heat in brine-based Li_2_CO_3_ production enables an additional 7–11% reduction in LFP and NMC811 cells’ climate change impacts by 2035 (Figure S3).

### 3.5 Limitations and Outlook

Our analysis is challenged by gaps in project-level data reporting and inherent uncertainties of future scenarios. An important source of uncertainty stems from the site-specific LCIs, as highlighted by the existing variability in the calculated impacts per site across the literature. This discrepancy reflects study-specific assumptions in LCI modeling (e.g., divergence on heat or freshwater use) that propagate to the characterized impacts. Prospective technological improvements were represented primarily through higher lithium recovery rates and reduced energy and reagent consumption. Other influential parameters such as the brine chemistry were kept constant in our model, although the lithium concentration may decline as the richest brine at a deposit is depleted. Lower lithium concentrations and higher impurity levels over time can increase environmental impacts since higher mass flows throughout the system will be needed to keep the same production yield.^30^ Future research should explore the constraints imposed by changes in brine chemistry on impact mitigation efforts. Operating projects may also adopt new technologies in the future, e.g., DLE technology adoption at existing sites such as Salar de Atacama. However, without detailed technical information, such changes remain speculative and were not incorporated into this study. In addition, mitigation strategies that are currently in a proof-of-concept stage may become viable in the future. CO_2_ captured either from on-site flue gas streams or via direct air capture (DAC) could be utilized as a feedstock in lithium brine processing, enabling the production of lithium carbonate while permanently storing CO_2_ through mineralization into solid carbonates. This approach represents a promising pathway that could simultaneously reduce energy-related CO_2_ emissions and decrease reliance on soda ash (thereby avoiding the impacts associated with its supply chain).^65,66^

Uncertainties also affect the supply mix-level impacts, here quantified on the basis of the production timelines of the 24 projects. This represents an incomplete list of projects as, e.g., the S&P database reports production by 2035 for 35 projects, although the 24 projects assessed in this study account for about 90% of the 2035 production. Yet, some projects may not materialize, and reported production volumes could be overly optimistic, as they often reflect company targets. Future research could build on this work as new site-specific data become available. Expanding the availability of site-specific LCIs remains crucial, with gaps remaining for sites in Argentina (e.g., Sal de Oro and Salta Lithium) and the United States (Fort Cady and Paradox).

Given the relevance of water use impacts in lithium supply, future research may place greater emphasis on minimizing freshwater consumption and improving water management and assessment practices. A still open methodological question concerns how brine water should be accounted for.^15^ In this study, brine evaporation was not included within freshwater consumption, as its high salinity does not meet the definition of freshwater in the AWARE method.^51^ This is a deliberate methodological choice, consistent with current practice in LCA. However, brine water is part of the catchment’s hydrological system, and its extraction can lower groundwater levels and draw in adjacent freshwater.^9^ Such effects are not specific to brine extraction but are common in mining more generally due to drainage activities and the removal of material from the underground.^67,68^ Under such a broader accounting framework, the water-scarcity impacts could be higher than those captured by focusing only on proces freshwater, and could differ across sites depending on local hydrogeology.^15^ Resolving how these indirect effects should be represented in water-scarcity assessments remains an active methodological question (see Section 4 in the Supporting Information). Moreover, we relied on static characterization factors, yet water scarcity could worsen in some regions over time, potentially resulting in higher characterization factors and impacts.^69^

Despite inherent limitations in modeling future scenarios, our bottom-up prospective LCA approach comprehensively integrates site-specific LCIs with future changes in production technologies and the broader economy, covering sectors such as reagent production overlooked before. This level of detail provides valuable insights into future drivers of environmental impact, supporting decision-making toward effective mitigation strategies with a life-cycle perspective and the benefit of foresight. With many expansion plans and new projects still under development, an important opportunity emerges to integrate mitigation measures early in the project life cycle, minimizing disruption and avoiding infrastructure lock-ins that could hinder impact reduction later. Operating projects may face greater constraints in adopting mitigation measures because of technical challenges and higher costs. While companies advance technology improvement, heat decarbonization must be prioritized, and the chemical reagent sector needs to accelerate the development of detailed decarbonization pathways. Moreover, lithium producers should adopt a supply-chain-wide approach to water management, prioritizing electricity and reagents with both low GHG emissions and minimal water footprints, which presents an added challenge in the pursuit of a more sustainable lithium supply chain.

## Supplementary Material





## Data Availability

The code to import the LCIs and perform the LCA calculations can be found at 10.5281/zenodo.22125927. To create the supply mix scenarios into a format suitable for use in prospective LCA through the *premise* framework, a Python library was created and made openly available for use with lithium and other commodities, provided users have a valid S&P database license (https://github.com/robyistrate/minerals_supply_scenarios).

## Notes

Figure 1: annual production of battery-grade lithium carbonate for the 24 projects disaggregated by country (a) and technology (b); Figure 2: life cycle climate change impacts of the 24 projects in 2025 and 2035 under alternative scenarios; Figure 3: life cycle water scarcity impacts of the 24 projects in 2025 and 2035 under alternative scenarios; Figure 4a: production-level life cycle climate change and water scarcity impacts of battery-grade Li_2_CO_3_ production under alternative scenarios disaggregated by country of the project; Figure 4b: production-level life cycle climate change and water scarcity impacts of battery-grade Li_2_CO_3_ production under alternative scenarios disaggregated by technology of the project; Figure 4c: production-level life cycle climate change and water scarcity impacts of battery-grade Li_2_CO_3_ production under alternative scenarios disaggregated by source of the impact; Figure 5: sensitivity of project-level life cycle climate change impacts to variations in soda ash and quicklime production by 2035; LCIs baseline: cumulative LCIs for each of the 24 projects under the baseline technological conditions; LCIs optimized: cumulative LCIs for each of the 24 projects under the optimized technological conditions (XLSX)
Additional information on the selection of brine-based lithium projects, source of site-specific LCIs, scenario modeling assumptions, and additional results (PDF)
