# Supplementary material for: Future Environmental Performance of Lithium from Brines Shaped by Energy and Reagent Decarbonization and Technology Optimization
Source: Environ Sci Technol. 2026 Sep 10;60(37):26408–20. doi: 10.1021/acs.est.6c09037 (PMC13614186; doi:10.1021/acs.est.6c09037)
Supplement: Supplementary file 1 [file es6c09037_si_002.pdf]

Supplementary Information

Future environmental performance of lithium from brines shaped by energy and reagent decarbonization and technology optimization

Robert Istrate,[1,*] Vanessa Schenker,[2] Antoine Beylot,[3] Victoire Collignon,[3] Stephan Pfister,[2] Bernhard Steubing[1]

[1] Institute of Environmental Sciences (CML), Leiden University, 2333 CC Leiden, The Netherlands
[2] Chair of Ecological Systems Design, Institute of Environmental Engineering, ETH Zurich, Zurich 8093, Switzerland
[3] BRGM, F-45060 Orléans, France
* Correspondence: i.r.istrate@cml.leidenuniv.nl

Contents

1. Selection of brine-based Li_2CO_3 production projects 2
2. Project-specific life cycle inventories modelling 5
    2.1. Project-specific electricity and heat sources 7
3. Scenarios modelling 11
    3.1. Country-specific electricity mixes 11
    3.2. Future changes in reagent production 13
        3.2.1. Quicklime 13
        3.2.2. Soda ash 15
        3.2.3. Sodium hydroxide 19
        3.2.4. Hydrochloric acid 19
    3.3. Future technology improvements 21
    3.4. Production-level scenarios 27
4. Water use impact assessment method 28
5. Additional results 30
6. References 36

1. Selection of brine-based Li_2CO_3 production projects

This study assesses the life cycle climate change and water scarcity impacts for 24 projects that produce battery-grade lithium carbonate (Li_2CO_3) from continental and geothermal brines (**Table S1**). As of April 2026, the S&P IQ Capital Pro database records 708 lithium projects, of which 145 focus on active lithium extraction from brine, identified by filtering for *"Deposit Type: Brine (Salar)"* under Geological Ore Body Type Zone 1 and "*Active*" in the activity status.[1] The database provides essential information for each project, including project name, primary product, location, development stage, activity status, projected start and closure years, etc. Whenever available, we supplemented gaps in brine-based projects details with information from other sources, such as company websites and reports.

The S&P database also provides project-specific production timelines through 2040. Here, we use production values from 2020 to 2035 as reported in the database where available. For projects without production data for 2035, we adopt a conservative approach: if the projected closure year is beyond the studied year (i.e., 2035), we assume the production volume remains constant from the previous year: if a project's closure year is after 2035, and production data for 2035 is missing, we estimate it based on the value for 2034. Projects without production data for 2025 and 2035 were excluded, resulting in a list of 35 projects. Therefore, 110 projects, or 76% of the projects contained in the dataset of all active projects, were discarded due to the lack of projected production data. Over 90% of these discarded projects are still in early exploration and prospecting stages (**Table S2**), explaining the limited information available on their future production. Finally, from the list of 35 projects with available production data, we considered a subset of 24 for which site-specific life cycle inventories (LCIs) were available.

The 110 projects excluded due to missing production volumes are in early development stages, making their plausibility of being operational by 2035 very limited, as project timelines often exceed 15 years from initiation to production.[2] Moreover, 11 projects with available production data were excluded due to missing LCIs: however, as demonstrated in Section 2.2.3 in the main manuscript, their contribution to total production would be relatively low. Therefore, despite the large number of projects discarded, our prospective analysis focuses on those with the highest likelihood of operating in the future.

For most projects, the output product is Li_2CO_3. If a project listed lithium brine concentrate or lithium hydroxide (LiOH) as the output product, we adjust the reported production volume to Li_2CO_3 by considering process losses. Specifically, for lithium brine concentrate, we assumed an overall efficiency of 80%, accounting for a 20% loss of lithium during purification, precipitation, and washing processes. This efficiency estimate is based on data from Salar de Atacama and is consistently applied across all projects due to the lack of site-specific data.[3] For LiOH production, we converted volumes to Li_2CO_3, assuming a 95% efficiency, reflecting a 5% loss of lithium during the conversion process from Li_2CO_3 to LiOH. Moreover, in some cases multiple output products are listed for the same project (e.g., Li_2CO_3 and lithium concentrate). For these projects, we assume that the entire reported production volume corresponds to Li_2CO_3. These assumptions introduce minimal uncertainty, as only a few projects produce something other than Li_2CO_3 or have multiple output products (**Table S1**). Moreover, we assume that all projects produce battery-grade Li_2CO_3 and that all the production stages were assumed to occur within the same country. This is the common practice as Li_2CO_3 is generally the commodity exported to other countries for usage or further refining into LiOH.

Table S1. List of 24 Li_2CO_3 production projects assessed in this study. Projects are ordered alphabetically. "ID" refer to the identification of the project in the S&P database. ET: Evaporitic technologies. DLE: Direct lithium extraction technologies. GEO: geothermal brine-based technology.

	Project name	ID	Country	Development stage	Product	Production data source	Technology category
1	Cauchari-Olaroz	37624	Argentina	Expansion	Carbonate	S&P database	ET
2	Chaerhan Lake	38549	China	Operating	Carbonate	S&P database	DLE
3	Cuenca Centenario-Ratones	52372	Argentina	Operating	Carbonate	S&P database	DLE
4	East Taijinair	38543	China	Operating	Carbonate	S&P database	DLE
5	Hombre Muerto North	79236	Argentina	Prefeas/Scoping	Carbonate	S&P database	ET
6	Kachi	78657	Argentina	Feasibility Complete	Carbonate	S&P database	DLE
7	Lakkor Tso Salt Lake	88960	China	Operating	Hydroxide	S&P converted to Li_2CO_3	DLE
8	Maricunga	38700	Chile	Construction Planned	Carbonate, Concentrate, Precipitates	Report value is assumed 100% Li_2CO_3 production	ET
9	Pastos Grandes	77807	Argentina	Construction Planned	Carbonate	S&P database	ET
10	PPG (Pozuelos-Pastos Grandes)	80115	Argentina	Prefeas/Scoping	Not specified	S&P assumed as Li_2CO_3	DLE
11	Qinghai Yiliping	88897	China	Commissioning	Carbonate	S&P database	DLE
12	Sal de los Angeles	37628	Argentina	Preproduction	Carbonate	S&P database	DLE
13	Sal de Vida	37611	Argentina	Construction Started	Carbonate	S&P database	ET
14	Salar De Arizaro	87450	Argentina	Prefeas/Scoping	Carbonate	S&P database	DLE
15	Salar de Atacama	37384 & 52581	Chile	Expansion	Carbonate, Chloride, Hydroxide, Sulfates	Report value is assumed 100% Li_2CO_3 production	ET
16	Salar de Olaroz	37059	Argentina	Expansion	Carbonate, Hydroxide	Report value is assumed 100% Li_2CO_3 production	ET
17	Salar de Tolillar	84909	Argentina	Prefeas/Scoping	Carbonate	S&P database	DLE
18	Salar del Hombre Muerto (Fenix)	37612	Argentina	Expansion	Carbonate, Chloride	Report value is assumed 100% Li_2CO_3 production	DLE
19	Salar del Rincon	36162	Argentina	Operating	Carbonate	S&P database	DLE
20	Silver Peak	37600	USA	Operating	Carbonate, Hydroxide	Report value is assumed 100% Li_2CO_3 production	ET
21	Tres Quebradas	78031	Argentina	Operating	Carbonate	S&P database	ET
22	Uyuni Salt Flat	36153	Bolivia	Construction Started	Carbonate	S&P database	DLE
23	Vulcan	83904	Germany	Commissioning	Hydroxide	S&P converted to Li_2CO_3	GEO
24	Zhabuye	38538	China	Expansion	Concentrate	S&P converted to Li_2CO_3	DLE

Table S2. Development stage of the projects discarded due to the lack of projected production data. These projects account for 76% of all active contained in the dataset.

Development stage	Stage description	No. discarded projects	Share discarded projects
Target Outline	Targets have been identified, and more detailed surface and/or underground exploration and drilling is under way*	45	41%
Exploration	Preliminary testing is underway (e.g., sampling, geochemical work, etc.)	29	26%
Reserves Development	Initial reserve/resource has been estimated	14	13%
Grassroots	Claims have been staked on prospects	9	8%
Prefeas/Scoping	Working on a preliminary assessment to determine mining and processing methods, and other projected economic metrics	9	8%
Limited Production	Some ore and/or commodity is being produced	2	2%
Preproduction	Go-aged decision has been made, and the project is being readied for production	1	1%
Satellite	Satellite of another project	1	1%
**Total**		**110**	**100%**

* https://rmis.jrc.ec.europa.eu/uploads/scoreboard2018/indicators/12._Mineral_exploration.pdf

2. Project-specific life cycle inventories modelling

Table S3 shows the brine-based Li_2CO_3 projects considered in this study alongside the source of the site-specific LCIs. LCI datasets for 21 projects were directly retrieved from Schenker & Pfister[4]. For further details on the model and the input parameters used the reader is referred to the original reference.[4] For the remaining three projects (East Taijinair, Lakkor Tso Salt Lake, and Zhabuye) the LCI dataset were developed in this work using the same model. The input parameters used to tune the model for these three projects are presented in **Table S4**. A summary of key LCI data for each project is provided in **Table S8**, while the full LCIs in a format readily available to be imported in Brightway are provided in the associated repository (https://doi.org/10.5281/zenodo.22125927).

Table S3. Sources of project-specific LCI datasets.

Project name	Country	LCI dataset
Cauchari-Olaroz	Argentina	Schenker & Pfister[4]
Chaerhan Lake	China	Schenker & Pfister[4]
Cuenca Centenario-Ratones	Argentina	Schenker & Pfister[4]
East Taijinair	China	This study
Hombre Muerto North	Argentina	Schenker & Pfister[4]
Kachi	Argentina	Schenker & Pfister[4]
Lakkor Tso Salt Lake	China	This study
Maricunga	Chile	Schenker & Pfister[4]
Pastos Grandes	Argentina	Schenker & Pfister[4]
PPG (Pozuelos-Pastos Grandes)	Argentina	Schenker & Pfister[4]
Qinghai Yiliping	China	Schenker & Pfister[4]
Sal de los Angeles	Argentina	Schenker & Pfister[4]
Sal de Vida	Argentina	Schenker & Pfister[4]
Salar De Arizaro	Argentina	Schenker & Pfister[4]
Salar de Atacama	Chile	Schenker & Pfister[4]
Salar de Olaroz	Argentina	Schenker & Pfister[4]
Salar de Tolillar	Argentina	Schenker & Pfister[4]
Salar del Hombre Muerto	Argentina	Schenker & Pfister[4]
Salar del Rincon	Argentina	Schenker & Pfister[4]
Silver Peak	USA	Schenker & Pfister[4]
Tres Quebradas	Argentina	Schenker & Pfister[4]
Uyuni Salt Flat	Bolivia	Schenker & Pfister[4]
Vulcan	Germany	Schenker & Pfister[4]
Zhabuye	China	This study

Table S4. Input data to the parametrized LCI model for Zhabuye, East Taijinair, and Lakkor Tso Salt Lake. This data was used as input into the parametrized model described in ref.[4]

	Zabuye	East Taijinair	Lakkor Tso Salt Lake
Region[a]	CN-NWG	CN-NWG	CN-NWG
Elevation	4421	2689	4506
Longitude	83.94731	93.94101	84.1355
Latitude	31.35917	37.52256	31.97135
**Brine chemistry (in wt. %)**			
Li	0.08	0.08	0.02
Cl	11.86	14.60	0.70
Na	9.71	0.00	0.93
K	0.00	1.44	0.16
Ca	0.00	0.02	0.00
Mg	0.38	2.92	0.05
SO_4	4.21	4.67	1.12
B	0.26	0.00	0.05
H_2O	73.50	76.27	96.97
Reference	Xu et al. (2021)[5]	Xu et al. (2021)[5]	Li et al. (2022)[6]
Enriched Li-conc.	1.07	-	-
**Further details**			
Distance to processing fac. (km)	10	-	-
Quicklime use reported	1 (Yes)	0 (No)	0 (No)
Second Li enrichment reported	0 (No)	0 (No)	0 (No)
Motherliquor reported	0 (No)	0 (No)	0 (No)
**Process sequence based on Schenker & Pfister[4]**			
process_1	evaporation_ponds	evaporation_ponds	evaporation_ponds
process_2	transport_brine	Mg_removal_quicklime	Li_adsorption
process_3	grinding	CentrifugeQuicklime	ion_exchange_L
process_4	washing_general	sulfate_removal_calciu mchloride	nanofiltration
process_5	Centrifuge_general	acidification	reverse_osmosis
process_6	dissolution	Li_adsorption	DLE_evaporation_ponds
process_7	Centrifuge_general	ion_exchange_L	Liprec_TG
process_8	ion_exchange_H	electrodialysis	CentrifugeTG
process_9	ion_exchange_H	reverse_osmosis	washing_TG
process_10	ion_exchange_L	Liprec_TG	dissolution
process_11	Liprec_BG	CentrifugeTG	Liprec_BG
process_12	CentrifugeBG	washing_TG	CentrifugeBG
process_13	washing_BG	dissolution	washing_BG
process_14	CentrifugeWash	Liprec_BG	CentrifugeWash
process_15	rotary_dryer	CentrifugeBG	rotary_dryer
process_16		washing_BG	
process_17		CentrifugeWash	
process_18		rotary_dryer	
Reference	Baiyin Zabuye Lithium Industry Co Ltd (2011)[7]	Ying et al. (2023)[8]	Zijin mining - Lakkor Tso Lithium Project (2025)[9]

Notes: [a] CN-NWG: Region according to ecoinvent - Northwest China Grid

2.1. Project-specific electricity and heat sources

Electricity and heat sources for each project were retrieved from technical reports and company disclosures whenever available. Overall, most projects rely on the electricity grid for power supply and use natural gas as the primary heat source (**Table S5**). In a few cases, electricity is generated on-site using natural gas engines (Pastos Grandes, Pozuelos-Pastos Grandes, Salar de Tolillar) or diesel generators (Hombre Muerto North, Sal de Vida, Salar de Arizaro). Diesel is also used as a heat source in some projects (e.g., Hombre Muerto North, Maricunga, Sal de Vida, Salar de Arizaro). Moreover, a few exceptions were identified: two projects report the use of electric boilers (Salar del Rincon) and propane (Silver Peak) for heat supply. When energy source could not be identified, we assumed the use of the national or regional electricity grid and natural gas for heat (e.g., as is the case for most Chinese projects).

To model the life cycle impacts of the electricity and heat sources, we used LCI datasets from the ecoinvent v3.101 (cut-off system model) database (or, in the prospective assessment, the LCIs as modified by *premise*) as follows:

- National or regional electricity grid: location-specific "*market for electricity, medium voltage*";
- On-site electricity generation with natural gas: "*electricity production, natural gas, conventional power plant*";
- On-site electricity generation with diesel: "*diesel, burned in diesel-electric generating set, 10MW*". It should be noted that lithium sites may rather use multiple smaller generators, yet such generators are not available in the ecoinvent database;
- Natural gas heat: "*heat production, natural gas, at industrial furnace >100kW*";
- Diesel heat: "*heat production, light fuel oil, at industrial furnace 1MW*" (used as a proxy);
- Propane heat: "*heat production, propane, at industrial furnace >100kW*";
- Electric boiler heat: LCI created considering only the input of electricity (source-specific) and assuming 99% efficiency;[10]
- On-site combined heat and power co-generation with natural gas: "*heat and power co-generation, natural gas, conventional power plant, 100MW electrical*";
- On-site combined heat and power co-generation with diesel: "*heat and power co-generation, diesel, 200kW electrical, SCR-NOx reductio*n".

Table S5. Project-specific electricity and heat sources assumed in the LCIs.

Project name	Reported power and heat sources	Modelling assumption		Source	Link to source
		Electricity source	Heat source		
Cauchari-Olaroz	Electricity: National grid (grid access at 60 km); backup diesel generator; solar energy potential highlighted<br>Heat: Natural gas	National grid	Natural gas	NI43-101 Operational Technical Report (2024)	https://minedocs.com/28/Cauchari-Olaroz-TR-12312024.pdf
Chaerhan Lake	Not available	Regional grid	Natural gas	Not available	Not available
Cuenca Centenario-Ratones	Not available	National grid	Natural gas	Not available	Not available
East Taijinair	Not available	Regional grid	Natural gas	Not available	Not available
Hombre Muerto North	Electricity: CHP operating with diesel<br>Heat: CHP operating with diesel	Diesel CHP	Diesel CHP	NI43-101 Preliminary Economic Assessment (2024)	https://www.lithiumsouth.com/wp-content/uploads/LIS_Preliminary_Economic_Assessment_-_HMN_Project_-_2024-04-24.pdf
Kachi	Electricity: National grid; minor solar PV; backup diesel generators<br>Heat: Not available	National grid	Natural gas	Definitive Feasibility Study (2025)	https://minedocs.com/30/Kachi-DFS-08042025.pdf
Lakkor Tso Salt Lake	Not available	Regional grid	Natural gas	Not available	Not available
Maricunga	Electricity: National grid<br>Heat: Diesel (to generate steam; switch to residual fuel oil or solar as alternatives in the future)	National grid	Diesel	NI43-101 Technical Report - Definitive Feasibility Study Update (2022)	https://lithiumpowerinternational.com/wp-content/uploads/2022/01/NI43-101_DFS2022_LR.pdf
Pastos Grandes	Electricity: Natural gas engines; minor PV supply<br>Heat: Natural gas; some heat produced with diesel due to limited natural gas supply capacity	Natural gas	Natural gas	NI43-101 Technical Report - Feasibility Study (2019)	https://minedocs.com/21/PASTOS-GRANDES-FS-07292019.pdf
PPG (Pozuelos-Pastos Grandes)	Electricity: Natural gas CHP<br>Heat: Natural gas CHP (supplying 65% of steam demand; remaining from conventional boiler)	Natural gas CHP	Natural gas CHP	NI43-101 Technical Report – Preliminary Economic Assessment (2019)	https://minedocs.com/11/PozuelosPastosGrandes_PEA_01172019.pdf
Qinghai Yiliping	Not available	Regional grid	Natural gas	Not available	Not available
Sal de los Angeles	Electricity: National grid (grid access at 100 km)<br>Heat: Natural gas (access at the same location as grid)	National grid	Natural gas	NI 43-101 Technical Report (2016)	https://s1.q4cdn.com/369274472/files/Sal-de-Los-Angeles-Technical-Report.pdf

Sal de Vida	Electricity: Diesel generators (off-grid power generation for isolated facilities; target 30% of power from solar PV)<br><br>Heat: Diesel boiler	Diesel	Diesel	NI 43-101 Technical Report (2022)	https://minedocs.com/22/SaldeVida_TR_03312022.pdf
Salar De Arizaro	Electricity: Diesel generators<br><br>Heat: Diesel boiler (opportunity for co-generation in the future)	Diesel	Diesel	NI 43-101 Technical Report – Pre-feasibility Study (2024)	https://lithiumchile.ca/wp-content/uploads/2024/10/Salar-de-Arizaro-NI-43-101-Technical-Report-and-PFS_FINAL-lith.pdf
Salar de Atacama	Electricity: National grid; diesel generator as backup; solar panels for minor activities<br><br>Heat: Natural gas	National grid	Natural gas	Technical report (2022)	https://minedocs.com/22/Salar-de-Atacama-TR-042022.pdf
Salar de Olaroz	Not available	National grid	Natural gas	Not available	Same assumption as Cauchari-Olaroz
Salar de Tolillar	Electricity: Gas-fired electricity generation plant<br><br>Heat: Not available	Natural gas	Natural gas	NI43-101 Technical Report – Preliminary Economic Assessment Update (2023)	https://minedocs.com/25/Tolillar-PEA-8102023.pdf
Salar del Hombre Muerto	Electricity: Not available<br><br>Heat: Natural gas	National grid	Natural gas	NS Energy project information	https://www.nsenergybusiness.com/projects/fenix-lithium-mine-salar-del-hombre-muerto/?cf-view&cf-closed
Salar del Rincon	Electricity: National grid<br><br>Heat: Electric boilers	National grid	Electric boiler	Environmental Impact Study (2024)	https://www.riotinto.com/en/operations/south-america/rincon
Silver Peak	Electricity: Regional grid (NV Energy Regional Transmission System)<br><br>Heat: Propane	Regional grid	Propane	Prefeasibility study (2025)	https://minedocs.com/19/Silver-Peak-TRS-06302024.pdf
Tres Quebradas	Electricity: Natural gas combined with solar panels at the salar site, and national grid at the purification site<br><br>Heat: Natural gas	National grid	Natural gas	NI 43-101 Technical Report (2021)	https://minedocs.com/21/Tres-Quebradas-FS-11252021.pdf
Uyuni Salt Flat	Not available	National grid	Natural gas	Not available	Not available
Vulcan	Electricity: National grid (produced electricity at the geothermal plants is exported to the grid)<br><br>Heat: Natural gas (produced heat from the geothermal brine is exported for regional district heating)	National grid	Natural gas	Environmental and Social Impact Assessment (ESIA) report (2024)	https://v-er.eu/app/uploads/2024/09/Vulcan-Zero-Carbon-Lithium-Project_ESIA-Report_Final_16092024_EN-3.pdf
Zhabuye	Electricity: Not available (solar PV combined with solar thermal and energy	Regional grid	Natural gas	Blooming Global news	https://www.bloomingglobal.com/media/detail/worlds-highest-zero-

	storage is mentioned in the Blooming reference attached, yet the specification of the system remains unclear)<br><br>Heat: Not available				carbon-lithium-project-launches-on-tibetan-plateau?utm_source=chatgpt.com

3. Scenarios modelling

3.1. Country-specific electricity mixes

Environmental impacts from electricity consumption in grid-connected projects were modelled using country-specific electricity mixes and their projected evolution between 2025 to 2035. **Table S6** shows the electricity mixes in 2025 and 2035 for the six lithium-producing countries considered in this study. The following paragraphs describe the data sources and assumptions for each country.

Table S6. National electricity mixes for Argentina, Bolivia, and Chile considered in this study in 2025 and 2035. Units: % of annual generation by power generation technology.

	Argentina		Bolivia		Chile	
	2025	2035	2025	2035	2025	2035
Coal	2%	–	–	–	18%	–
Natural gas	52%	23%	62%	20%	15%	2%
Oil	4%	0%	1%	–	0.1%	0.3%
Bioenergy	2%	2%	3%	2%	6%	1.6%
Nuclear	7%	9%	–	–	–	–
Hydro	19%	28%	28%	40%	22%	11%
Wind	12%	25%	2%	14%	13%	58%
Solar PV	3%	13%	3%	20%	25%	27%
Geothermal	–	–	–	–	–	0.2%
Other	–	–	–	–	–	–

Electricity mix of Argentina

The electricity mix of Argentina in 2025 was obtained from the Ember Electricity Data Explorer[b], showing a mix highly dominated by natural gas-fired power generation, which accounts for 52% of total electricity generation, with hydropower (19%) and wind power (12%) representing the most relevant renewable energy sources. Projections for 2035 were based on the report *Lineamientos y Escenarios para la Transición Energética a 2050*, published by the Argentinean Ministry of Economy.[c] This report outlines three long-term energy pathways for the country: the *Base* scenario, which reflect the continuation of current policies; the *Optimista* scenario, which incorporates additional policy measures and technological developments; and the *Ambicioso* scenario, which assumes a more accelerated adoption of decarbonization technologies. All three scenarios are designed to be consistent with Argentina's NDC targets. For this study, the *Base* scenario was selected to represent the electricity mix by 2035. Under this scenario, the share of natural gas-fired electricity generation decreases substantially, declining from 52% in 2025 to 23% by 2035. At the same time, substantial expansion of renewable electricity generation capacity is projected, particularly for hydropower, wind power, and solar PV. Additional hydropower investments, including the commissioning of 2.5 GW by 2030, are expected to increase hydropower's share of the electricity mix from 19% in 2025 to 28% by 2035. Wind and solar PV capacity are also projected to expand considerably, reaching 25% and 13% of the electricity mix by 2035, respectively. Nuclear power generation is expected to increase modestly as well, from 7% in 2025 to 9% in 2035, due to the addition of 700 MW by 2030.

[b] Available at https://ember-energy.org/data/electricity-data-explorer/

[c] Available at https://www.boletinoficial.gob.ar/detalleAviso/primera/289827/20230707

Electricity mix of Chile

The electricity mix of Chile in 2025 was obtained from the Ember Electricity Data Explorer[d], showing a highly diversified mix composed primarily of solar PV (25% of the mix), hydropower (22%), coal (18%), natural gas (15%), and wind power (13%). Future projection for 2035 is based on the scenarios developed in the Chilean Government's *Planificación Energética a Largo Plazo* (PELP)[e]. The PELP defines three long-term energy pathways for Chile: *Recuperación Lenta*, *Carbono Neutralidad*, and *Transición Acelerada*. Among these, the *Carbono Neutralidad* scenario was selected for this study because it is aligned with Chile's NDC targets. Under this scenario, Chile is expected to completely phase out coal-fired power generation and substantially reduce natural gas power generation by 2035. Most of the planned capacity expansion over the next decade is concentrated in wind power, with additions of 4.2 GW by 2026, 7.5 GW by 2029, and 6.1 GW by 2035. As a result, wind power is projected to account for 58% of the generation mix by 2035 from just 13% in 2025. Some additional solar PV capacity is also expected, increasing its share of generation from 25% in 2025 to 27% in 2035. In contrast, while hydropower generation is projected to remain relatively stable in absolute terms (from approximately 20 TWh in 2025 to 17 TWh by 2035), its contribution to the electricity mix is expected to decline from 22% in 2025 to 11% in 2035 due to the rapid growth of wind and solar PV.

Electricity mix of Bolivia

The electricity mix of Bolivia in 2025 was obtained from the Ember's Electricity Data Explorer[f], reflecting a mix dominated by natural gas (62% of the mix) and hydropower (28%). The projection of by mix by 2035 is based on the report "*Plan Eléctrico Referencial del Estado Plurinacional de Bolivia 2035*" published by the Ministerio de Hidrocarburos y Energía of Bolivia in September 2025.[g] The expansion of the electricity grid presented in the plan is based on the target of 65% renewable power generation by 2029 and 75% by 2035 considering current and announced power generation projects in the country. The Bolivia electricity mix expects an increase in the share of hydropower generation from 28% in 2025 to 40% by 2035, primarily due to the large 600 MW Rositas project expected to enter production by 2032 (cf. Table 9 in national plan). The shares of wind and solar PV are also expected to increase from 2% to 14% and from 3% to 20% between 2020 and 2035, respectively, as many projects are expected to enter operation over the next decade with capacities ranging from 50 to 104 MW for wind and from 30 to 120 MW for solar. In addition to hydropower, wind, and solar, the 100 MW Potosí geothermal project is expected to enter operation by 2033, representing approximately 4% of the mix by 2035. The increase in renewable power generation capacity will mainly affect natural gas generation, which share in the mix will decrease from 62% by 2025 to 20% by 2035.

Electricity mix of China, USA, and Germany

Electricity mixes for China, USA, and Germany are based on the REMIND SSP2-NDC scenario.[11] For Germany, the electricity mix corresponds to the Western Europe mix, as the REMIND scenario data used in this work do not disaggregate Europe into countries.

[d] Available at https://ember-energy.org/data/electricity-data-explorer/

[e] Available at https://energia.gob.cl/pelp/proyecciones-electricas

[f] Available at https://ember-energy.org/data/electricity-data-explorer/

[g] Available at https://www.mhe.gob.bo/wp-content/uploads/2025/10/PLAN-ELECTRICO-REFERENCIAL-2035-FINAL.pdf

3.2. Future changes in reagent production

The most relevant reagents used in battery-grade Li_2CO_3 production from brines are quicklime, soda ash, hydrochloric acid, and sodium hydroxide.[4] We considered future changes in the production of these reagents by focusing on the main hotspots for GHG emissions and water scarcity. Thus, changes that may affect other impact categories, such as the implemented of air pollution control systems driven by more stringent air pollution emissions targets, have been omitted.

Given the limited availability of information on future production pathways for these reagents, our projections primarily rely on estimates of energy and raw material consumption and emissions levels based on Best Available Techniques (BAT). We assume that production processes will progressively reach BAT performance levels in the future. The following subsections provide detailed narratives of the scenarios and the underlying assumptions.

3.2.1.Quicklime

Quicklime is produced through calcination of limestone followed by sizing and screening to obtain milled quicklime.[12] The process is highly carbon-intensive, emitting 1.0–1.8 kg CO_2-eq per kg.[13] About 65% of these emissions are related to chemical reactions during limestone calcination and 35% stem from fuel combustion for heating provision.[14] Therefore, the main decarbonization options for the lime industry involves process optimization and energy efficiency to reduce the demand for raw materials and fuel, fuel switching, and carbon capture and storage (CCS).[13,15–17] The main option for optimizing the process and improving energy efficiency is by switching from horizontal kilns to more efficient vertical kilns.[15,16] For example, the share of horizontal rotating kilns in Europe is gradually decreasing due to energy costs and high emission levels.[15,16] In this work, we considered production based on the most-efficient vertical parallel flow regenerative kiln (PFRK), the most popular kiln nowadays.[13] Limestone washing before transport to the quicklime plant is the main contributor to water scarcity impacts, followed by minor contributions from background processes such as electricity generation and fuels supply.[18] We do not account for future changes in limestone supply, while changes in the water scarcity impacts of some background processes may be affected by changes in the sectors modified by premise (e.g., electricity generation). The following paragraphs describe the LCIs used to model quicklime production as well as the assumptions made to integrate future changes regarding (i) process optimization and energy efficiency improvements, (ii) fuel switch, and (iii) CCS adoption.

<u>Life cycle inventories for quicklime production</u>

The LCIs for quicklime production in a PFRK without and with CCS have been compiled from the European Commission's Best Available Techniques (BAT) Reference Document for the production of lime,[19] Laveglia et al.,[12] and Foteinis et al.[20] The LCI consider the preparation of limestone, limestone calcination, and grinding of quicklime to finer grades. We do not consider the washing of limestone before feeding into the kiln, as this is not common practice (less than 10% of lime plants wash the limestone according to the BAT Reference Document).[19] Moreover, we do not model the hydration of quicklime (slaking) required to produce slaked lime since our final product is just quicklime (i.e., water consumption for the slaking process is not considered).

We obtained average consumption of raw materials and energy and waste generation for quicklime production in a PFRK from the BAT Reference Document, using the average values to represent the current situation.[19] About 1.8 kg of limestone are feed into the kiln to produce 1 kg of quicklime. However, the input of limestone to plant is slightly higher since some of it is rejected during preparation due to, e.g., low quality. Limestone fines can account for 1–5% of the annual production.[19] By considering an average of 3%, we estimated that 1.85 kg of limestone enters the plant to produce 1 kg of quicklime. From this, 0.05 kg is rejected as inert waste (fines limestone) and the remaining 1.8 kg is fed into the kiln. The kiln

operation consumes 3.7 MJ heat kg^{-1} quicklime and 0.055 kWh electricity kg^{-1} quicklime.[19] CO_2 emissions from limestone decomposition are based on stoichiometry, resulting in 0.785 kg CO_2 kg^{-1} quicklime.[12] Infrastructure requirements where modelled as $1{\cdot}10^{-12}$ unit of factory and $1{\cdot}61^{-5}$ kg of machinery per kg of quicklime based on Foteinis et al.[20] We further completed the LCI with data from ecoinvent v3.10.1 for diesel burned on-site for transportation and small quantities of water emissions. Air pollutants and other emissions resulting from the combustion of fuels (e.g., NO_x, SO_x, and PM) are considered in the corresponding ecoinvent LCIs used to model heat supply.

The LCI for the option with CCS considers that the lime kiln is retrofitted to integrate oxyfuel combustion followed by CCS, as described in Foteinis et al.[20] Oxygen originates from air separation, requiring an additional consumption of 0.073 kWh electricity kg^{-1} quicklime. CO_2 purification and capture is achieved through an improved cryogenic separation and distillation system, which requires about 0.090 kWh electricity kg^{-1} quicklime.[20] Only CO_2 from limestone calcination is captured. The captured CO_2 (0.785 kg CO_2 kg^{-1} quicklime) is assumed to be compressed, transported, and stored. We modelled these processes with an LCI from premise.[21]

<u>Process optimization and energy efficiency improvements</u>

The input of limestone to plant can be reduced by minimizing losses through the use of higher quality limestone, improved limestone preparation, and optimized kiln techniques that can operate with a wider range of limestone grain sizes.[19] We assumed that further improvements in the future will reduce limestone rejection rate to 1% (lower bound from the BAT Reference Document),[19] reducing the input amount of limestone to plat from 1.818 kg per kg quicklime. Note that the net amount of limestone feed into the lime kilns stays constant at 1.800 kg. Therefore, the stoichiometric amount of CO_2 resulting from limestone decomposition does not change over time.

In this work, we considered production based on the most efficient PFRK, thereby future improvements in energy efficiency are rather limited. Options to improve energy efficiency at the kiln level include waste heat use, efficient insulation, optimal combustion conditions, and optimal maintenance.[15,16] However, improvement opportunities in PFRK are modest as these kilns are already operated close to the heat of reaction.[15,16] For example, the average thermal efficiency of lime production in Europe reaches 88%.[15] We assumed that further energy efficiency measures in the near-term can lower heat consumption by around 14% by 2035, corresponding to the minimum value of 3.2 MJ heat kg^{-1} quicklime reported in the BAT Reference Document.[19] Moreover, measures will be taken to reduce electricity consumption, which can decrease by 45% by 2035 based on the minimum BAT value of 0.03 kWh electricity kg^{-1} quicklime.[12,16] In the PFRK with CCS, electricity consumption reduction is more modest, only 12% due to the higher electricity demand associated with CO_2 capture and purification.

<u>Fuel switch</u>

The fuel mix used in lime kilns can vary by kiln type and region. In Europe, about 53% of the fuel consist of solid fossil fuels (hard coal, lignite, and petroleum coke), 34% is natural gas, 8% waste, 3% oil, and 2% biomass (latest data available for year 2019).[12,16] Australia, for example, shows similar shares: 50% coal, 43% natural gas, 4% waste, 2% biomass, and <1% oil.[22] Efforts within the industry focuses on reducing the share of solid fossil fuels. For example, Laveglia et al. assessed several scenarios for Europe considering 20–100% reduction in solid fossil fuels by 2050 in favor of natural gas and biomass.[12] The European Lime Association identifies a complete shift from fossil solid fuels to natural gas as the most relevant decarbonization strategy in the near-term.[16]

Due to the lack of global data on the fuel mix in lime kilns, we assumed the European fuel mix to be representative of the current situation. For the future, we assumed the optimistic scenario from Laveglia et al., which assumes a full elimination of coal and oil by 2050, an increase in the share of natural gas and

biomass to 60% and 32%, respectively, and a constant 8% share of waste.[12] We note that a complete shift to natural gas may be challenged by infrastructure issues, such as the availability of gas pipeline in the area of the kilns.[17] Moreover, assuming a globally uniform fuel mix is a simplification. As lime is a low-cost material, typically produced locally with limited long-distance transport,[19] the fuel mix can vary considerably across production sites. The use of electricity for kiln heating has not been considered, as its economic feasibility remains uncertain.[16]

CCS adoption

CCS for lime kilns does not appear fully economically feasible in the near-term.[16] Other barriers involve logistics and infrastructure for transporting captured CO_2 from lime plants, usually located next to quarry and not in industrial clusters, to the injection and storage site.[16] The European Lime Association roadmap assumed that only some large kilns may be equipped with CCS by 2050.[16] However, in the UK several lime kilns aims at implementing CO_2 capture by 2030–2035.[23] Therefore, in this work we assume a moderate share of production from kilns equipped with CCS: 10% by 2030 and 15% by 2035.

3.2.2. Soda ash

Soda ash (sodium carbonate) is primarily produced either from trona mineral and sodium carbonate-rich brines (natural soda ash) or from salt brine and limestone via the Solvay process (synthetic soda ash). For natural soda ash, only trona is of commercial importance, with commercially exploitable deposits occurring only in the US, Turkey, and China.[24] In 2024, synthetic soda ash accounted for 67% of global production, while trona soda ash accounted for the other 33% (**Figure S1**).[25] Soda ash is produced in two grades, dense and light soda ash. Dense soda ash is produced through dehydration of light soda ash, and it is used mainly in the glass industry.[26] Here we modelled light soda ash grade suitable for use in minerals processing.

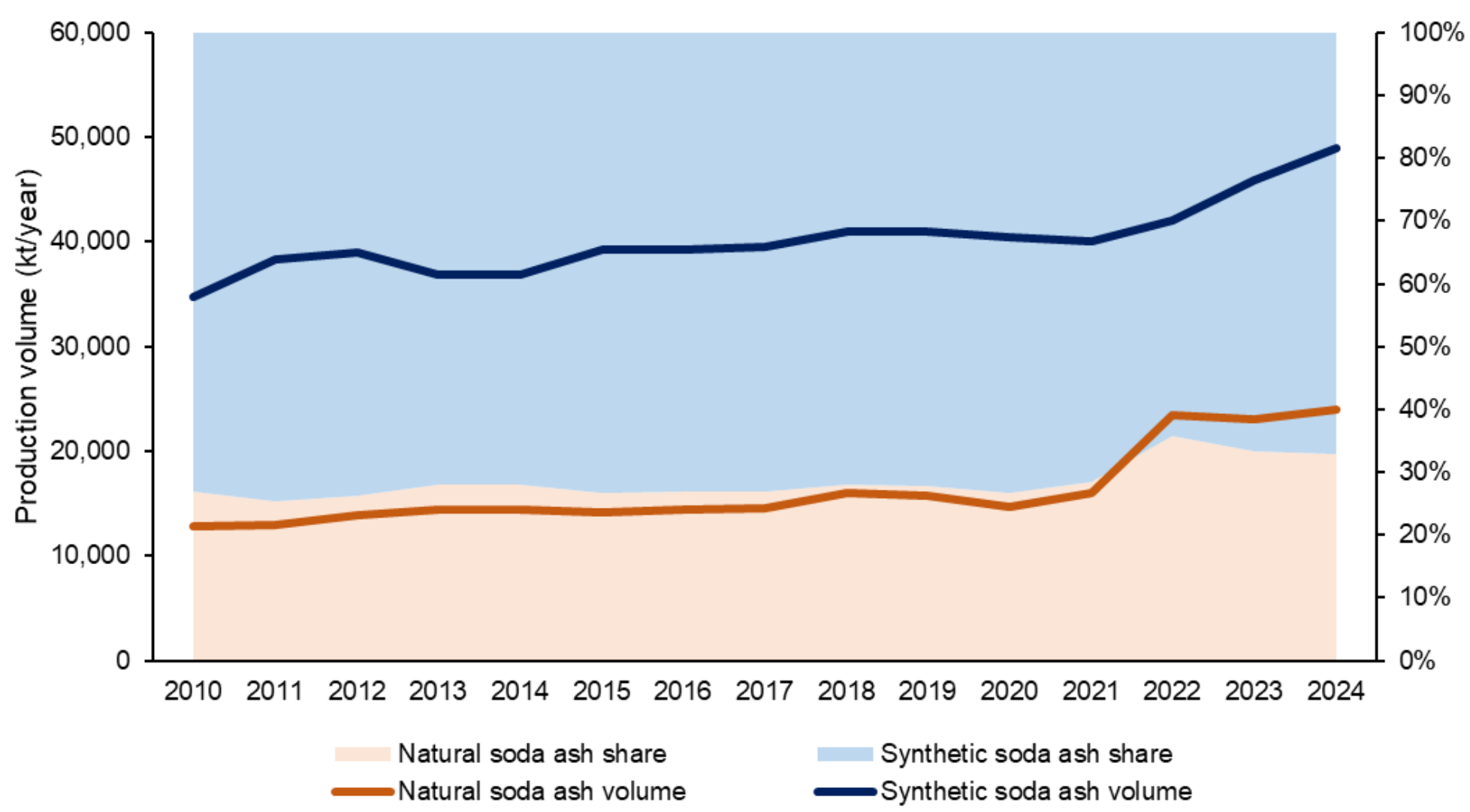

Figure S1. Trona and synthetic soda ash production volumes (right y-axis and solid lines) and market shares (left y-axis and stacked area) over 2010-2024. Source: USGS.[25]

Trona soda ash has lower carbon footprint and production costs than the synthetic route, and its market share has increased moderately over time, from 27% in 2010 to 33% in 2024 (**Figure S1**).[27] In this work, we assume that the observed growth in the market share of trona soda ash will continue in the future.[28] The main sources of CO_2 emissions in the trona-based production process arise from the calcination of trona ore, as well as from fuel combustion in crushers, driers, and boilers.[29] Synthetic soda ash is produced through the reaction of sodium chloride, CO_2, ammonia, and water. The CO_2 for the reaction is obtained

by limestone calcination with coke, while the quicklime produced in the process is used to regenerate ammonia (which achieve an almost total regeneration and recycle).[26] Being applied for more than 150 years, the Solvay process can be considered as a fully optimized process.[30] However, it still require large amounts of raw materials and steam,[26] which production is a major source of GHG emissions, and generate significant direct process emissions of CO_2.[31] Further optimization to reduce raw materials and heat demand, fuel switching to reduce reliance on fossil fuels, and the integration of CCS represent key decarbonization strategies for the natural and synthetic soda ash industry. These options are therefore integrated into our future projections.

<u>Life cycle inventories for soda ash production</u>
The LCI for the synthetic route (Solvay process) is based on the European Commission's BAT Reference Document on large volume inorganic chemicals, using the average values to represent the current situation.[26] The modelled system consider the following production stages: i) purification of the brine to remove impurities such as calcium and magnesium, ii) limestone screening and lime calcination and slaking, iii) absorption of ammonia (including ammonia recovery), iv) precipitation and separation of sodium bicarbonate, and v) calcination of sodium bicarbonate to produce light soda ash.[26]

The total consumption of limestone at the plant equals 1.30 kg per kg of soda ash (BAT range of 1.1–1.5 kg). The generation of fines of limestone was assumed to account for 0.2 kg per kg of soda ash (BAT range of 0.05-0.35 kg), equivalent to 15% of the input limestone. Sodium chloride consumption is equal to 1.6 kg per kg soda ash (BAT range of 1.5–1.7 kg), while the quantity of ammonia required for the make-up is relatively negligible at 0.0015 kg per kg soda ash (BAT range of 0.0008 – 0.0021 kg) due to the high ammonia recovery rate. Coke consumption in the lime kiln equals 2.32 MJ per kg soda ash (BAT range of 2.04-2.59 MJ), heat from steam 7.6 MJ per kg soda ash (BAT range of 6.11 to 9.17), and electricity consumption 0.09 kWh per kg soda ash (BAT range of 0.05–0.13 kWh). Excess CO_2 from limestone calcination with coke in the range of 0.2 to 0.3 kg is vented to the atmosphere, from which we used the average to represent current situation. Air pollutants and other emissions resulting from the combustion of fuels (e.g., NO_x, SO_x, and PM) are considered in the corresponding ecoinvent LCIs used to model heat supply.

The Solvay process is water intensive. The BAT Reference Document reports water intensities per kg of soda ash (dense) in the range 2.5–3.6 kg process water and 50–100 kg cooling water.[26] For the process water, 1.9–2.4 kg are used in the production of lime milk, which does not require high quality water and can be recycled from the cooling water system. On the other hand, the remaining 0.6–1.2 kg requires high purity water for washing purposes, and is assumed to come as freshwater input. Discharged water includes liquid effluent from the distillation of 8.5–10.7 kg per kg soda ash. Cooling water is largely recirculated as process water. Industry data reports water intensities for all processes, boiler, and cooling equal to 8.75 kg kg^{-1} trona soda ash.[32]

The Solvay process has two main co-products in addition to soda ash: calcium chloride and sodium bicarbonate per kg of soda ash.[26] Solving this multifunctionality would require allocating the LCI flows among the three co-products. However, it should be noted that the recovery of calcium chloride is not so popular due to its low demand. In fact, most of the calcium chloride is discarded with the wastewater discharge.[33] Therefore, we treat calcium chloride as a waste rather than a co-product. In contrast, sodium bicarbonate production is often integrated within the soda ash facility, and needs to be treated as a co-product.[26]

The LCI for the modified Solvay process with CCS is adapted from Rumayor et al.[34] The CO_2 from the soda ash direct gas emission stream is captured through absorption using monoethanolamine (MEA), which is

an established CO_2 capture technology achieving a capture efficiency of 90%. CO_2 capture requires about 5 g of MEA per kg of CO_2 that enters the system, equivalent to 0.43 g MEA kg^{-1} soda ash. Moreover, the CO_2 capture requires 4.26 MJ of heat for solvent regeneration and 0.13 kWh of electricity for operating fans and pumps as well as for compression and dehydration of the captured CO_2.[34]

The LCI for trona soda ash production is largely based on primary data from production facilities in the US.[29,35] The LCI aggregates the following generic production stages: (i) mining, screening, and crushing of trona ore; (ii) calcination of the crushed ore, resulting in anhydrous sodium carbonate and insoluble impurities and releasing CO_2 and water; (iii) dissolution of calcined trona in water resulting in a sodium carbonate solution; (iv) filtration of the sodium carbonate solution and multistage evaporative crystallization; (v) recovery of soda ash as monohydrate crystals via centrifuges and drying to remove the water, producing granular dense soda ash.[35]

An overall heat demand of 2.8 MJ kg^{-1} trona soda ash has been obtained from the US EPA, representing average production in the US.[29] Other raw materials and energy inputs were obtained from the USLCI database,[35] which includes averages from five plants in the US. According to this data source, producing 1 kg of trona soda ash requires about 0.12 kWh electricity, 0.013 MJ of fuel (assumed to be diesel) combusted in machinery and industrial equipment, minor inputs of raw materials, (e.g., quicklime, activated carbon, cement, or steel), and the disposal of 0.06 kg of inert waste and 0.16 kg of non-sulfidic tailings. We omitted some minor raw materials and transportation reported in the original LCIs, as these are expected to be negligible for the overall climate change and water scarcity impacts. The calcination of trona ore generates a stoichiometric amount of 0.138 kg CO_2 kg^{-1} soda ash.[29] Air pollutants and other emissions resulting from the combustion of fuels (e.g., NO_x, SO_x, and PM) are considered in the corresponding ecoinvent LCIs used to model heat supply.

The trona soda ash route is less water intensive than the Solvay process. Industry data reports water intensities for all processes, boiler, and cooling ranging from 1.6 to 2.4 kg kg^{-1} trona soda ash.[32] However, it is unclear if this range also includes the mining and processing of trona ore. For example, the USLCI dataset, which includes all stages, reports a higher water consumption of 4.13 kg kg^{-1} trona soda ash. Therefore, we consider as a conservative estimate the intensity reported in the USLCI dataset. Moreover, this source indicates that about 22% of the input water is discharged into water bodies.[35]

Production routes market shares

We assume that the market share of natural soda ash produced from trona will increase further in the future following historical trends over the period 2010–2024.[28] For example, no major capacity expansions of synthetic soda ash are being considered in Europe, while trona mining in the US is expected to expand considerably.[28] However, it is unlikely that trona soda ash capacity will expand quickly due to the lengthy process of mining permitting and implementations.[28] Thus, in our scenario, trona soda ash would reach 38% market share by 2035 while synthetic soda ash would decrease to 62% (**Figure S2**). It should be noted that the production share between natural and synthetic soda ash varies regionally. For example, the unavailability of trona deposits in Europe (excluding Turkey) makes that 100% of its soda ash production follows the Solvay process,[26] while the US has a mix of trona and synthetic soda ash production.[29] However, we do not disaggregate production by region due to the lack of data.

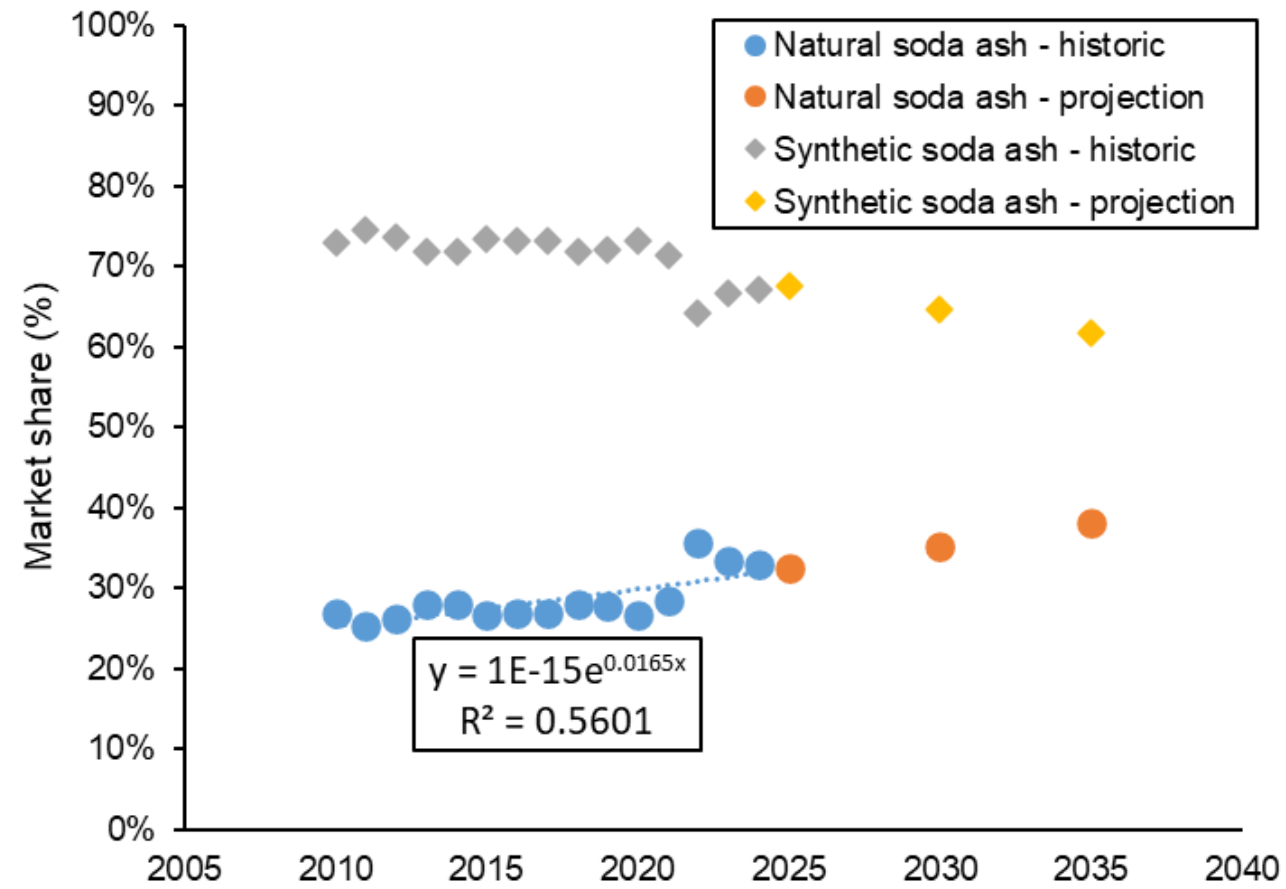

Figure S2. Projection of natural and synthetic soda ash market shares to 2035 based on historical data.

Process optimization

In the near future, further optimization of the Solvay process will result in lower consumption of raw materials (limestone and sodium chloride) and energy (coke, heat, and electricity). We based this reduction projections on the minimum values reported in the European Commission's BAT Reference Document.[26] Accordingly, consumptions per kg of soda ash would decrease by 2035 by 25% for limestone, 8% for sodium chloride, 12% for coke, 20% for heat/steam, and 44% for electricity.

Water intensity may decrease in the future according to companies' reported goals of optimizing water usage and increasing recycling. Solvay announced in 2020 plans to reduce freshwater intake by 25% by 2030 compared to 2018 through water use efficiency improvement and recycling.[36] Similarly, the Wyoming's trona industry in the US has announced efforts to cut water intensity by 15% through eliminating discharges and maximizing water recycling and reuse.[37] In this study, we assumed that these reductions in water intensity could be achieved by 2035.

Fuel switch

Fuels are used for calcination of trona ore, calcination of limestone in the lime kiln, and to produce steam for the Solvay process. Coke is used as fuel in the lime kiln due to the necessity to obtain high CO_2 concentration. Thus, switches to other fuels such as natural gas would result in too low CO_2 concentrations, and were not considered in the future.[26]

Heat for the calcination of trona ore or steam for the Solvay process are typically obtained by burning coal, natural gas, or fuel oil.[26,29] There is limited information available on the fuel mix used in trona and synthetic soda ash production globally. According to US EPA data from 2009, the US fuel mix for trona ore calcination consists of about 60% coal, 39% natural gas, and 1% fuel oil. In a more recent article from 2023, Scott indicated that about three-quarters (75%) of synthetic soda ash producers depend on coal for their processes.[28] The Solvay company, the world's largest soda ash producer, operates seven soda ash plants worldwide (both trona and synthetic), five of them being powered by coal.[38] These findings suggest a relatively high share of coal in the fuel mix used in soda ash production, yet exact numbers are largely missing. Therefore, in this work we assume that for the current situation 60% of the heat supplied to synthetic soda ash production comes from coal and 39% from natural gas, and a minor 1% from biomass, omitting fuel oil due to its relatively negligible contribution.

Soda ash companies plan to cut GHG emissions primarily through replacing coal boilers with biomass.[28,39] Solvay's global sustainability goals include commitment to completely phase-out coal for energy supply

before 2030.[38] In this work, we assume that the share of coal in the fuel mix will decrease to a residual 5% by 2035. The gap left by coal phasing-out will be partially filled by biomass to some extent, reaching 20% of the fuel mix by 2035. Natural gas will act as a transitioning fuel, reaching 75% of the fuel mix by 2035. We applied the same fuel mix to both trona and synthetic soda ash production.

CCS adoption

CCS has been studied as an alternative for reducing direct CO_2 emissions from the Solvay process.[30,34,40,41] For example, Krótki et al. demonstrated the feasibility CO_2 capture through a pilot plant integrated with an existing soda ash production facility from Qemetica Soda Polska S.A. in Poland.[40] Due to the lack of projections on CCS adoption for the Solvay process, we make the same assumption as in the case of quicklime, assuming a modest 15% of production with CCS by 2035.

3.2.3. Sodium hydroxide

About 99% of global sodium hydroxide is co-produced with chlorine in the chlor-alkali process, which involves the electrolysis of a sodium chloride brine.[42] The current global electrolysis technology mix consists of about 83% membrane cell, 15% diaphragm cell, and 3% mercury cell.[18] More than 95% of the life cycle GHG emissions of sodium hydroxide are associated with electricity consumption in the electrolysis process.[43] According to the European Commission's BAT Reference Document on the chlor-alkali process, mercury cells have the highest electricity requirements at 3.0–4.4 kWh electricity per kg chlorine, with membrane cell requiring the least at 2.3–3.0 kWh per kg chlorine, relatively similar to diaphragm cell at 2.6–3.1 kWh per kg chlorine.[42] Moreover, water scarcity impacts are mainly associated with water losses in the electrolysis process (about 50%) and electricity generation (about 30%). Notably, the diaphragm technology has relatively higher water consumption compared to the other two technologies.[42] Therefore, in this work we consider a progressive shift towards the more efficient membrane cell technology in the future.

Electrolysis technologies market shares

At present, membrane cell technology is dominant, mercury cells are being phased out, and only limited new diaphragm cell capacity is being added.[43] Technology roadmaps indicate a continued shift toward membrane cell technology. For example, in Europe the share of diaphragm technology declined from about 20% in 2001 to 8.4% in 2023, while mercury cells have been prohibited since 2017.[44] A decarbonization roadmap for the Dutch chlor-alkali industry propose the large-scale implementation of membrane cell technology as the most relevant strategy to reduce electricity consumption and associated GHG emissions.[45] Therefore, the more efficient membrane cell technology is expected to fully replace the other technologies in the future.[46] In this work, we assume a complete transition to membrane cells by 2035 (membrane cell accounting for 100% of the production mix).

3.2.4. Hydrochloric acid

Industrial-grade hydrochloric acid (30% concentrated) is typically produced by dissolving hydrogen chloride in water.[47] Various pathways exist for obtaining the hydrogen chloride precursor. Based on the ecoinvent v3.10 database, more than 95% of hydrochloric acid is produced via four main routes: (i) the Mannheim process (~40%), (ii) combustion of hydrogen with chlorine (~40%), (iii) as a by-product of methylene diphenyl diisocyanate production (<10%), and (iv) as a by-product of toluene diisocyanate production (<10%).[18] Global production data disaggregated by pathway are not available; therefore, we assumed a constant technology mix over time as reported in ecoinvent. However, we note that more than half of the life cycle GHG emissions of hydrochloric acid are attributable to electricity use, primarily from chlorine production in the chlor-alkali process. Chlorine production is also responsible for about 40% of the water scarcity impact, with diaphragm technology contributing most significantly (see sodium hydroxide above for further

details). The Mannheim process represents another major source of water scarcity, due to water losses within the process itself as well as in the production of its precursors, sulfuric acid and sodium chloride.

We assessed future impacts of hydrochloric acid production by considering changes in the underlying chlorine production technology mix. Consistent with the assumptions applied to sodium hydroxide production (see above), we assumed a complete phase-out of diaphragm and mercury cells technologies in favour of the more efficient membrane cell technology by 2035.

3.3. Future technology improvements

To assess future environmental impacts in the context of technology improvement (*2035 +Optimized* scenario), we rely on the prioritized parameters defined in Schenker & Pfister.[4] We defined the following rules to account for technological improvements (**Table S7**):

1. **Overall lithium recovery rate:** The lithium recovery rates are changed in two ways, as the literature often discusses potential high recovery rates for DLE technologies and relatively low recovery rates for conventional evaporitic technologies.[48] Thus, the recovery rate for DLE technologies increases from 77% to 95% while the recovery rate for conventional evaporitic technologies increases by 20% relative to the baseline assumption.
2. **Efficiency of processes:** Efficiencies used in processes are increased to a maximum.
3. **Chemical surpluses:** Chemicals, such as soda ash, quicklime, and hydrochloric acid, are calculated using stoichiometry and a surplus to account for the incompleteness of the reaction. In this scenario, the surplus is reduced to <1 %.
4. **Lithium enrichment factors:** Higher lithium concentrations of the effluent of processes, such as Li-adsorption and evaporation ponds are used to reduce subsequent mass flows.
5. **Electricity reduction of devices:** All devices are updated and require 90 % of the electricity demand used in the default scenario.

Table S8 provides an overview of the energy and reagents demand and water flows associate with the baseline and optimized LCIs.

Table S7. Overview of key technology parameters under the baseline and optimized assumptions. Parameters which value change from the baseline to the optimized case are highlighted in orange.

Parameter	Unit	Baseline	Optimized
Bond working index (Grinding)	-	13	13
CO_2/process-specific output (Dissolution)	kg/kg	10	1
Ca-concentration (Ca-Mg removal by sodium hydroxide)	wt.%	0.0002	0.0002
Chlorine emissions/process-specific output (Ion exchanger)	kg/kg	-0.00023	-0.00023
Heat demand/ process-specific output (Evaporator)	MJ/kg	145	145
Hydrochloric acid demand/process-specific output (Ion exchanger)	kg/kg	0.00024	0.00024
Initial lithium concentration (Reverse osmosis)	ppm	2500	2000
Lithium concentration (Evaporation ponds after DLE)	wt. %	2.3	4.37
Lithium concentration of effluent (Reverse osmosis)	ppm	5000	10000
Lithium concentration of effluent (Adsorption)	ppm	10000	19000
Lithium concentration of effluent (Evaporator)	ppm	30000	40000
Mg concentration of pulp (Mg removal by quicklime)	wt.%	0.05	0.05
Sodium hydroxide demand/process-specific output (Ion exchanger)	kg/kg	0.00012	0.00012
Sodium emissions/process-specific output (Ion exchanger)	kg/kg	-0.00007	-0.00007
Temperature (Electrodialysis)	°C	25	25
Temperature (Ion exchanger)	°C	70	70
Temperature (Lithium carbonate precipitation)	°C	83	83
Temperature (Mg removal by soda ash)	°C	60	60
Temperature (Reverse osmosis)	°C	40	40
Temperature (Adsorption)	°C	85	85
Temperature (Boron removal by org. solvent)	°C	10	10
Temperature (Desorption)	°C	40	40
Temperature (Li_2CO_3 dissolution)	°C	10	10
Temperature (Mechanical evaporator)	°C	70	70
Temperature (Mother liquor recycled)	°C	80	80
Temperature (Nanofiltration)	°C	45	45
Adsorption capacity (Geothermal brines)	kg/kg	0.008	0.0591
Adsorption capacity (Salar brines)	kg/kg	0.024	0.0591
Electricity demand (Belt filter)	kWh/kg	0.4	0.36
Calcium chloride solution (dil. in water)		0.3	0.3
Centrifuge (battery grade) output (mix Li_2CO_3 + water)		1.5	1.5
Ratio liquid waste/output (Centrifuge battery grade)	-	-1	-0.1
Ratio output/produced Li_2CO_3 (Centrifuge technical grade)	-	1.5	1.5
Ratio water recycled/output (Centrifuge technical grade)	-	0.2	0.2
Ratio liquid waste/output (Centrifuge technical grade)	-	-0.8	-0.1
Electricity demand (Centrifuge)	kWh/kg	0.01	0.009
Ratio water demand/produced Li_2CO_3 (Washing)		1.5	1.5
Discarded washing water after centrifuge (Centrifuge)		-1	-0.1
Dissolution constant of LiCl in aqueous solution (Dissolution)	g/L	15	15
Efficiency of wells	%	95	97
Grinding efficiency (Grinding)	%	80	80

Electricity demand/process-specific output (Ion exchanger)	kWh/kg	0.00082	0.00074
Electricity demand/process-specific output (Evaporator)	kWh/kg	2	1.8
Electricity demand/process-specific output (Nanofiltration)	kWh/kg	8.25	7.43
Electricity demand/process-specific output (Reverse osmosis)	kWh/kg	2.78	2.50
Electricity demand/process-specific output (Adsorption)	kWh/kg	0.00074	0.00066
Gain output ratio (Evaporator)		16	16
Ratio steam input/process-specific output (Evaporator)		0.2	0.2
Final grain size (Grinding)	μm	75	75
Heat demand/process-specific output (Ion exchanger)	MJ	-0.00162	-0.00162
Heat loss	%	15	15
Initial grain size (Grinding)	μm	1000	1000
Ratio input/process-specific output (Nanofiltration)	-	0.05	0.05
Ratio mother liquor/input of Mg removal process	-	5	0.5
pH after acidification	-	1.8	6
pH before acidification	-	11	8
Freshwater use/process-specific output (Evaporation ponds based on Atacama)	kg/kg	0.0027	0.0027
Harvested mass of salt/process-specific output (Evaporation ponds based on Atacama)		8.57E-05	8.57-05
Moisture content of harvested salt	%	10	10
Used quicklime/process-specific output (Evaporation ponds based on Olaroz)	kg/kg	0.041	0.008
Salt to carbonate ratio	-	0.15	0.15
Quicklime reaction factor to account for reaction incompleteness	-	1.2	1
Recycling rate of organic solvent (Boron removal)	%	98.5	98.5
Removal fraction (Electrodialysis)	%	90	90
Electricity demand/process-specific output (Rotary dryer)	kWh/kg	1	0.9
Heat demand/process-specific output (Rotary dryer)	MJ/kg	0.3	0.3
Percentage of heat waste/process-specific output (Rotary dryer)	%	50	50
Salinity (Electrodialysis)		1	1
Soda ash reaction factor to account for reaction incompleteness	-	1.3	1
Soda ash solution (dil. in water)		0.25	0.25
Sodium hydroxide reaction factor to account for reaction incompleteness	-	1.2	1
Sodium hydroxide solution (dil. in water)		0.3	0.3
Sulfuric acid solution (dil. in water)		0.18	0.18
Required mass of water/process-specific output (Washing)	kg/kg	2	1
Ratio waste/process-specific output (Washing)	kg/kg	0.1	0.1
Waste/process-specific output (Grinding)	kg/kg	0.1	0.1
Water/process-specific output (Ion exchanger)	kg/kg	1.1	1.1
Required water for desorption/mass of resin	kg/kg	100	10
Required water covering evaporation ponds/initial mass in evaporation ponds	kg/kg	0.1	0.1
Electricity demand for water purification	kWh/kg	2.783	2.783
Water input/overall water demand due to enhanced recycling	kg/kg	0.25	0.25
Waste/overall water demand due to enhanced recycling	kg/kg	0.25	0.25
Water demand/process-specific output (Grinding)	kg/kg	1.5	1.5
Hours of excavator working in evaporation ponds	hours/kg	37	37

Table S8. Overview of energy, reagents, and water flows per kg of battery-grade Li_2CO_3 for the 24 projects in the baseline and optimized life cycle inventories (LCIs). Baseline LCIs are used to model the *2025* and *2035 Background* scenarios while the optimized LCIs are used in the *2035 +Optimized* and *2035 +ElecHeat* scenarios.

LCI flow	Electricity		Heat		Quicklime		Soda ash		Sodium hydroxide		Steam		Sulfuric acid		Wastewater		Process water, evaporation		Process water, withdrawal	
**Scenario**	**Baseline**	**Optimized**	**Baseline**	**Optimized**	**Baseline**	**Optimized**	**Baseline**	**Optimized**	**Baseline**	**Optimized**	**Baseline**	**Optimized**	**Baseline**	**Optimized**	**Baseline**	**Optimized**	**Baseline**	**Optimized**	**Baseline**	**Optimized**
**Units**	**kWh**	**kWh**	**MJ**	**MJ**	**kg**	**kg**	**kg**	**kg**	**kg**	**kg**	**kg**	**kg**	**kg**	**kg**	**kg**	**kg**	**kg**	**kg**	**kg**	**kg**
Salar de Atacama	1.43	1.29	18	13	0.02	0.01	2.05	1.66	0.03	0.05	0.00	0.00	0.03	0.03	3.81	4.44	46	51	58	54
Salar de Cauchari-Olaroz	1.61	1.22	24	13	2.75	2.70	1.87	1.44	0.08	0.10	0.08	0.09	0.00	0.00	2.21	2.71	34	32	69	39
Salar de Olaroz	2.16	2.02	9.06	8.61	3.67	0.73	1.86	1.43	0.02	0.02	0.00	0.00	0.11	0.11	7.16	7.88	96	102	160	154
Silver Peak	1.91	1.77	8.17	7.05	0.36	0.07	1.86	1.43	0.02	0.02	0.00	0.00	0.00	0.00	1.22	1.94	28	33	84	79
Tres Quebradas	2.06	1.89	36	19	0.00	0.00	1.93	1.50	0.06	0.08	0.00	0.00	0.04	0.04	4.60	5.44	55	61	66	61
Chaerhan	11	2.42	358	292	0.00	0.00	1.86	1.43	0.09	0.00	0.00	0.00	0.10	0.08	7.53	6.99	119	82	245	95
East Taijinar	5.26	1.80	205	83	1.38	0.57	1.86	1.43	0.09	0.00	0.00	0.00	0.07	0.03	5.76	4.38	103	55	254	80
Fenix	5.69	2.23	269	209	0.00	0.00	7.90	6.07	0.04	0.00	4.30	0.17	0.06	0.06	11	6.00	194	76	287	105
Lakkor Tso	12	2.86	478	388	0.00	0.00	1.86	1.43	0.09	0.00	0.00	0.00	0.13	0.11	9.33	8.45	139	99	267	114
Salar de Centenario	5.86	2.16	286	193	0.00	0.00	10	6.64	0.04	0.00	4.30	0.17	0.07	0.06	12	5.70	201	73	304	104
Salar del Rincon	5.94	2.22	314	215	0.00	0.00	10	6.66	0.04	0.00	4.30	0.17	0.08	0.06	12	5.94	205	76	308	107

LCI flow	Electricity		Heat		Quicklime		Soda ash		Sodium hydroxide		Steam		Sulfuric acid		Wastewater		Process water, evaporation		Process water, withdrawal	
**Scenario**	**Baseline**	**Optimized**	**Baseline**	**Optimized**	**Baseline**	**Optimized**	**Baseline**	**Optimized**	**Baseline**	**Optimized**	**Baseline**	**Optimized**	**Baseline**	**Optimized**	**Baseline**	**Optimized**	**Baseline**	**Optimized**	**Baseline**	**Optimized**
**Units**	**kWh**	**kWh**	**MJ**	**MJ**	**kg**	**kg**	**kg**	**kg**	**kg**	**kg**	**kg**	**kg**	**kg**	**kg**	**kg**	**kg**	**kg**	**kg**	**kg**	**kg**
Zhabuye	1.74	1.53	12	10	12	1.48	0.00	0.00	0.02	0.02	0.00	0.00	0.00	0.00	1.34	2.02	30	35	83	74
Maricunga	3.07	2.22	47	22	0.21	0.06	2.49	2.09	0.06	0.04	0.00	0.00	0.13	0.13	11	12	152	145	251	168
Sal de Vida	1.34	1.22	13	11	0.35	0.34	1.88	1.45	0.00	0.00	0.00	0.00	0.00	0.00	2.20	2.78	28	32	41	37
Salar de Pastos Grandes	3.23	3.09	17	15	6.18	6.17	1.95	1.51	0.09	0.11	0.00	0.00	0.08	0.08	7.35	8.05	87	93	106	103
Salar del Hombre Muerto North	1.75	1.52	48	22	3.37	3.19	1.91	1.48	0.08	0.11	0.00	0.00	0.05	0.05	7.11	8.55	79	91	76	71
Kachi	7.90	3.74	367	290	0.00	0.00	12	7.98	0.04	0.00	4.30	0.17	0.11	0.09	14	7.44	228	95	342	132
Pozuelos	6.82	2.87	431	354	0.00	0.00	14	9.16	0.04	0.00	4.30	0.17	0.13	0.10	15	8.32	242	106	365	149
Qinghai Yiliping	11	2.42	342	279	0.00	0.00	1.86	1.43	0.09	0.00	0.00	0.00	0.10	0.08	7.53	6.99	119	82	245	95
Sal de los Angeles	6.37	2.56	375	299	0.00	0.00	7.35	4.86	0.04	0.00	4.30	0.17	0.11	0.09	14	7.63	226	94	320	119
Salar de Arizaro	6.36	2.50	320	233	0.00	0.00	20	13	0.04	0.00	4.30	0.17	0.08	0.07	12	6.29	219	86	361	141
Salar de Tolillar	7.05	3.54	314	276	0.00	0.00	9.13	7.02	0.04	0.00	4.30	0.17	0.08	0.08	13	7.20	209	91	308	125
Salar de Uyuni	5.93	2.12	206	100	0.00	0.00	29	19	0.04	0.00	4.30	0.17	0.03	0.03	9.56	3.91	195	65	372	141

LCI flow	Electricity		Heat		Quicklime		Soda ash		Sodium hydroxide		Steam		Sulfuric acid		Wastewater		Process water, evaporation		Process water, withdrawal	
**Scenario**	**Baseline**	**Optimized**	**Baseline**	**Optimized**	**Baseline**	**Optimized**	**Baseline**	**Optimized**	**Baseline**	**Optimized**	**Baseline**	**Optimized**	**Baseline**	**Optimized**	**Baseline**	**Optimized**	**Baseline**	**Optimized**	**Baseline**	**Optimized**
**Units**	**kWh**	**kWh**	**MJ**	**MJ**	**kg**	**kg**	**kg**	**kg**	**kg**	**kg**	**kg**	**kg**	**kg**	**kg**	**kg**	**kg**	**kg**	**kg**	**kg**	**kg**
Upper Rhine Graben	17	2.13	165	12	0.43	0.42	1.87	1.43	0.28	0.00	1.22	0.00	0.00	0.00	112	33	0.00	0.00	445	39

3.4. Production-level scenarios

We used the development stage of projects to develop two production-level scenarios (*Operating* and *Operating+Announced*). The definition of these development stages is provided in **Table S9**.

Table S9. Project development stages in the S&P Database and their consideration on our production-level scenarios.

Development stage	Description	Operating	Operating+Announced
Early-stage	Project without resource estimate		
Grassroots	Claims have been staked on prospects		
Exploration	Preliminary testing is underway (e.g., sampling, geochemical work, etc.)		
Target Outline	Targets have been identified, and more detailed surface and/or underground exploration and drilling is under way		
Late-stage	Project with a defined resource that has not yet reached a production decision		
Reserves development	Initial reserve/resource has been estimated		●
Advanced exploration	Drilling activities to add additional reserves/resources		●
Prefeasibility/Scoping	Working on a preliminary assessment to determine mining and processing methods, and other projected economic metrics		●
Feasibility			●
Feasibility started	Feasibility report has commenced		●
Feasibility completed	Feasibility report is complete		●
Mine-Stage			
Preproduction	Go-aged decision has been made and the project is being readied for production	●	●
Construction planned	Construction is planned		●
Construction started	Construction has started		●
Commissioning	Mine is commissioned and production has started		●
Operating	Project is fully operational	●	●
Satellite	Satellite of a another project	●	●
Expansion		●	●
Limited production	Some ore and/or commodity is being produced	●	●
Residual production			
Closed	Operation has stopped		

4. Water use impact assessment method

In this work, life cycle water scarcity impacts were quantified using the Available WAter REmaining (AWARE) method, a consensus-based midpoint method developed within the UNEP-SETAC Life Cycle Initiative.[49] AWARE quantifies the potential to deprive humans and ecosystems of water when freshwater is consumed in a given region. It is based on the relative availability of water remaining per unit of area in a region once the demand of humans and aquatic ecosystems has been met.[49] Water scarcity impacts of producing battery-grade lithium carbonate at a given project p (WSI_p) were obtained by multiplying the volume of freshwater consumed over the life cycle (FW_p) by the corresponding AWARE characterization factor (CF):

$$WSI_p = FW_p \times CF$$

The AWARE CF represents the relative water scarcity impact of consuming 1 m^3 in a given area compared with the world-average cubic meter consumed (m^3_{world}-eq/m^3). The CF ranges from 0.1 to 100, where a value of 1 corresponds to a region whose remaining water per area and time equals the world average, values below 1 indicate regions that are less water-scarce than the world average, and a value of 100 corresponds to a region where the water remaining per area and time is 100 times lower than the world average.[49] AWARE provides CFs at basin, country, and global levels. We used basin-level, non-irrigation CFs to quantify the impacts of foreground processes (**Table S10**) and the global-average, unspecified CF to quantify impacts linked to background processes.

Freshwater consumption (FW_p) refers to water removed from a watershed and no longer available to it, for example through evaporation or incorporation into products. As AWARE is defined for freshwater,[49] and given that the high mineral content of brine makes it unsuitable for humans and most aquatic ecosystems,[50] brine water evaporation was not included in the calculation of FW_p in this work.

Whether brine water evaporation should be accounted for in water scarcity impact assessments has led to diverging approaches in the LCA literature, with some authors including it in the freshwater consumption calculation[51–53] and others excluding it.[4,54] The main argument for excluding brine is that its salinity prevents its direct use by humans or aquatic ecosystems, so it does not meet the definition of freshwater underlying the AWARE method.[50] The main argument for including it is that brine is part of the hydrological system of a catchment, and that its extraction at a salar can alter the surrounding hydrological regime by drawing in adjacent water.[55] Such effects are not specific to brine extraction but are common in mining more generally due to drainage activities[56] and the removal of material from the underground.[57] Through these mechanisms, brine extraction may have an indirect effect on freshwater availability.[55,58] Consequently, while there is little disagreement that brine itself is not usable freshwater, no consensus has yet emerged on whether and how the hydrological effects of brine extraction should be reflected in LCAs.[58] Yet, site-specific hydrological studies are better suited for representing these indirect effects and should inform further impact assessment methods development in LCA.

Table S10. AWARE CFs used for each project. Site-specific CF from the AWARE method considering all water consumption sectors except irrigation and agriculture.

Project name	Country	CF (m^3_{world}-eq/m^3)
Cauchari-Olaroz	Argentina	5.00
Chaerhan Lake	China	70.6
Cuenca Centenario-Ratones	Argentina	2.70
East Taijinair	China	28.4
Hombre Muerto North	Argentina	2.70
Kachi	Argentina	4.80
Lakkor Tso Salt Lake	China	92.6
Maricunga	Chile	100
Pastos Grandes	Argentina	100
PPG (Pozuelos-Pastos Grandes)	Argentina	100
Qinghai Yiliping	China	100
Sal de los Angeles	Argentina	2.70
Sal de Vida	Argentina	2.70
Salar De Arizaro	Argentina	8.20
Salar de Atacama	Chile	94.7
Salar de Olaroz	Argentina	5.00
Salar de Tolillar	Argentina	2.70
Salar del Hombre Muerto	Argentina	2.70
Salar del Rincon	Argentina	11.0
Silver Peak	USA	91.7
Tres Quebradas	Argentina	96.5
Uyuni Salt Flat	Bolivia	25.1
Vulcan	Germany	0.70
Zhabuye	China	92.6

5. Additional results

This section presents additional results not included in the main manuscript:

- **Figure S3** shows the correlation between the contribution of electricity, heat, and reagents to climate change impacts and the impact mitigation across scenarios.
- **Figure S4** shows the relative change in climate change and water scarcity impacts in 2035 compared to 2025 for background processes supplying electricity, reagents, and heat supply.
- **Figure S5** compares the relative change in climate change impacts in the *2035 +ElecHeat* scenario relative to the *2035 +Optimized* scenario when low-temperature heat electrification is implemented using heat pumps or electric boilers.
- **Figure S6** shows the correlation between the contribution of electricity, heat, reagents, and on-site water consumption to water scarcity impacts and the impact change across scenarios.
- **Figure S7** shows life cycle climate change and water scarcity impacts of LFP and NMC811 cells production in China in 2025 and 2035 using the lithium supply mix from this study. For the production of the battery cells, we used the LCIs from ecoinvent v3.10.1, modified by *premise* to account for increasing cell energy density.

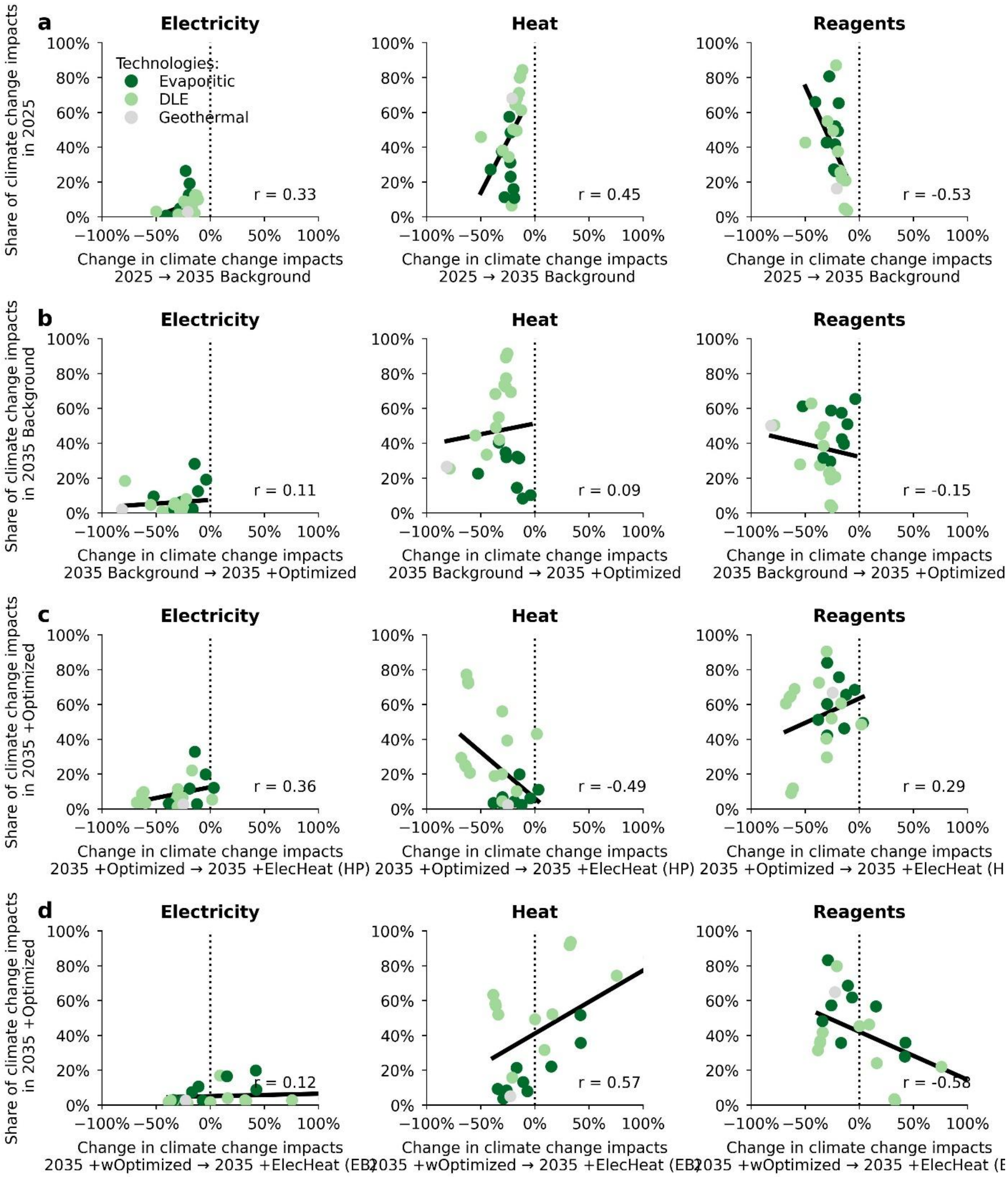

Figure S3. Correlation between the contribution of electricity, heat, and reagents to climate change impacts and the impact mitigation across scenarios. The y-axis shows the contribution of each source to climate change impacts in the *2025* (a), *2035 Background* (b) and *2035 +Optimized* (c, d) scenarios. The x-axis shows the relative change in climate change impacts between scenarios: (i) from *2025* to *2035 Background*, (ii) from *2035 Background* to *2035 +Optimized*, (iii) from *2035 +Optimized* to *2035 +ElecHeat* with heat pumps (HP), and (iv) from *2035 +Optimized* to *2035 +ElecHeat* with electric boilers (EB). Negative values indicate impact reduction. Each point represents one project (n = 24)

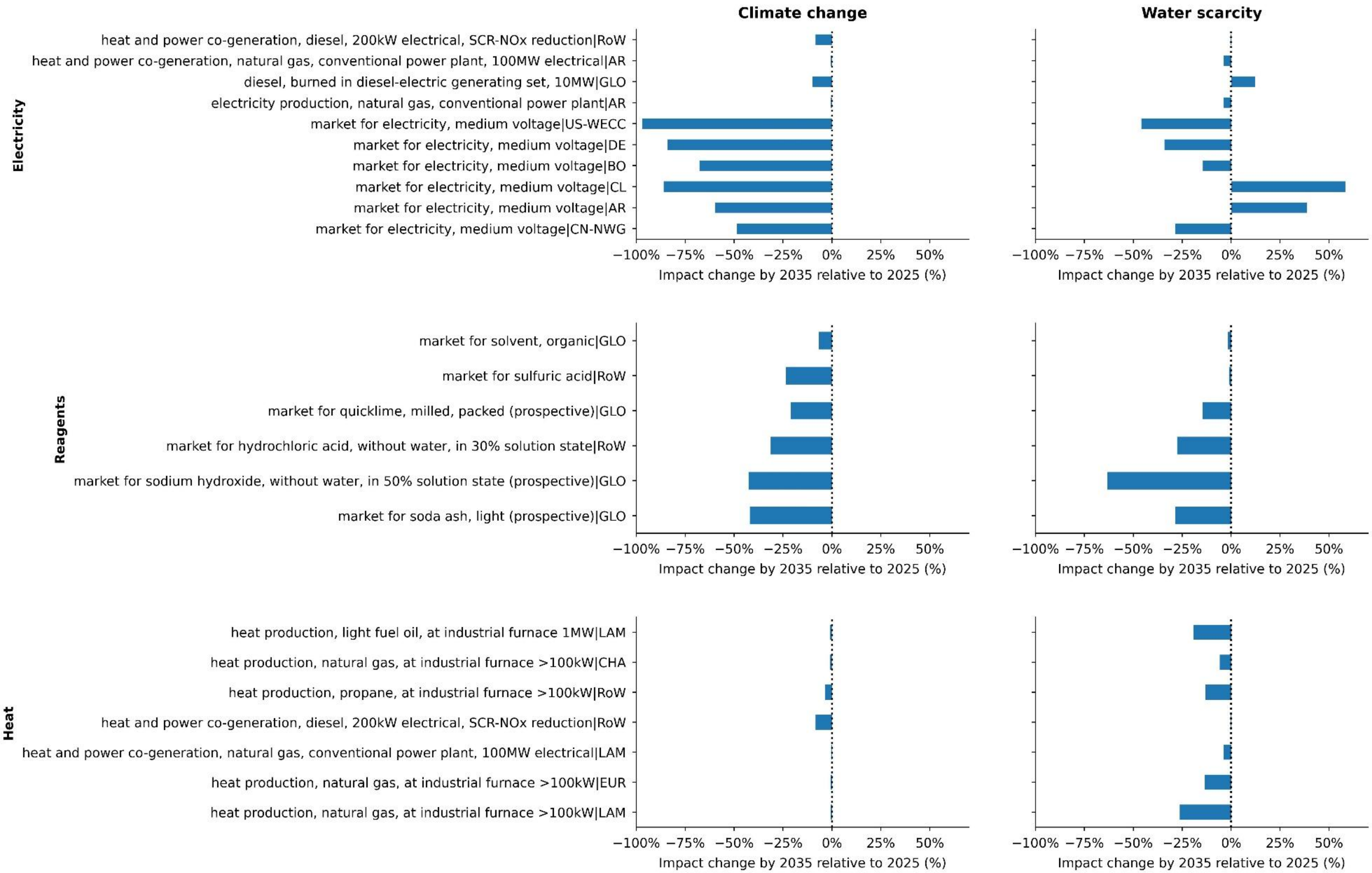

Figure S4. Relative change in climate change and water scarcity impacts in 2035 compared to 2025 for background processes supplying electricity, reagents, and heat. Negative values indicate a reduction in impacts relative to 2025, while positive values indicate an increase. The labels in the y-axis indicate the name of the background process (based on ecoinvent) alongside the geographical location.

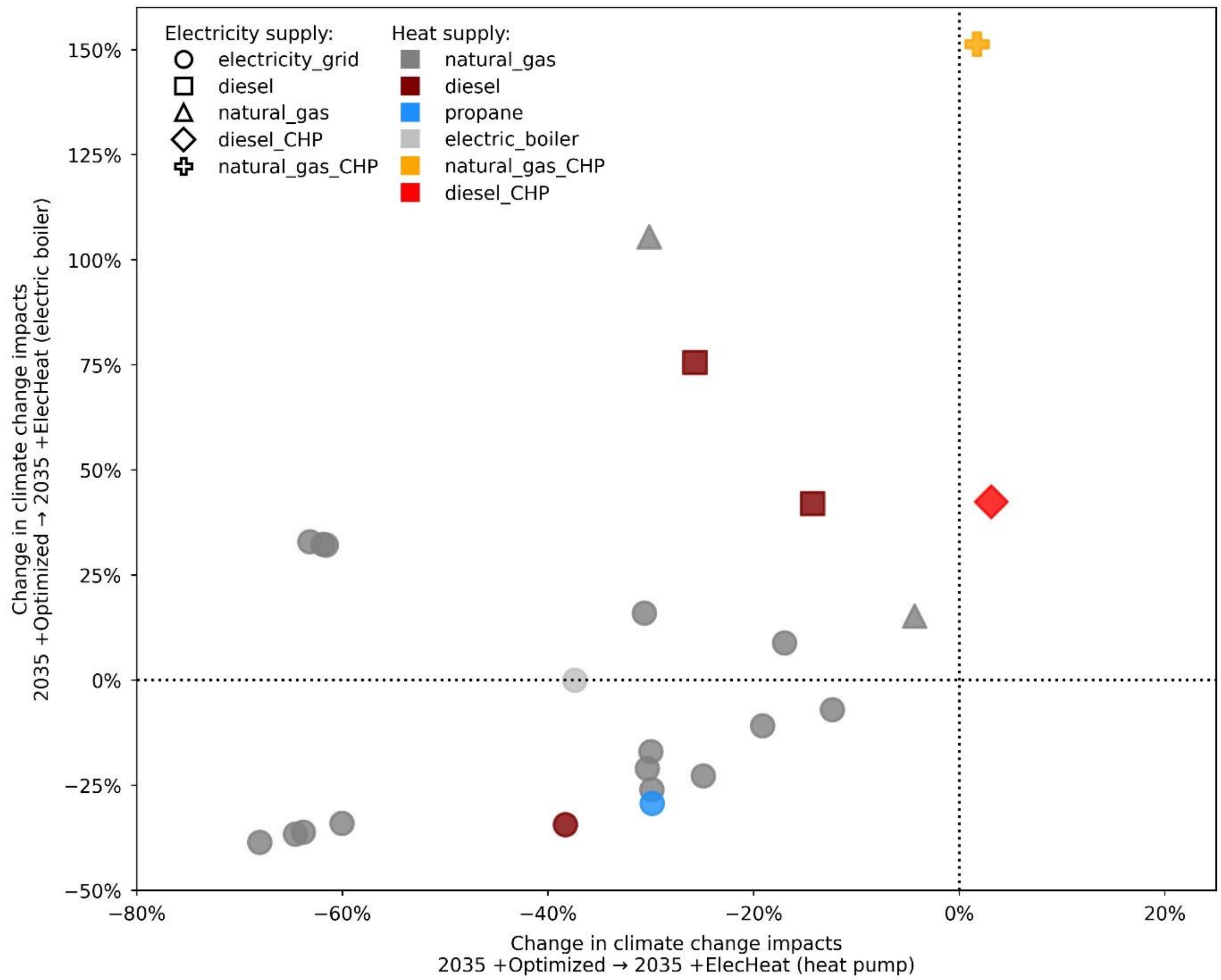

Figure S5. Relative change in climate change impacts in the *2035 +ElecHeat* scenario relative to the *2035 +Optimized* scenario when low-temperature heat electrification is implemented using heat pumps (*x*-axis) or electric boilers (*y*-axis). Negative values indicate impact reduction. Each point represents one project (n = 24). Marker shapes denotes project-specific electricity supply source, while colors indicate the initial heat source.

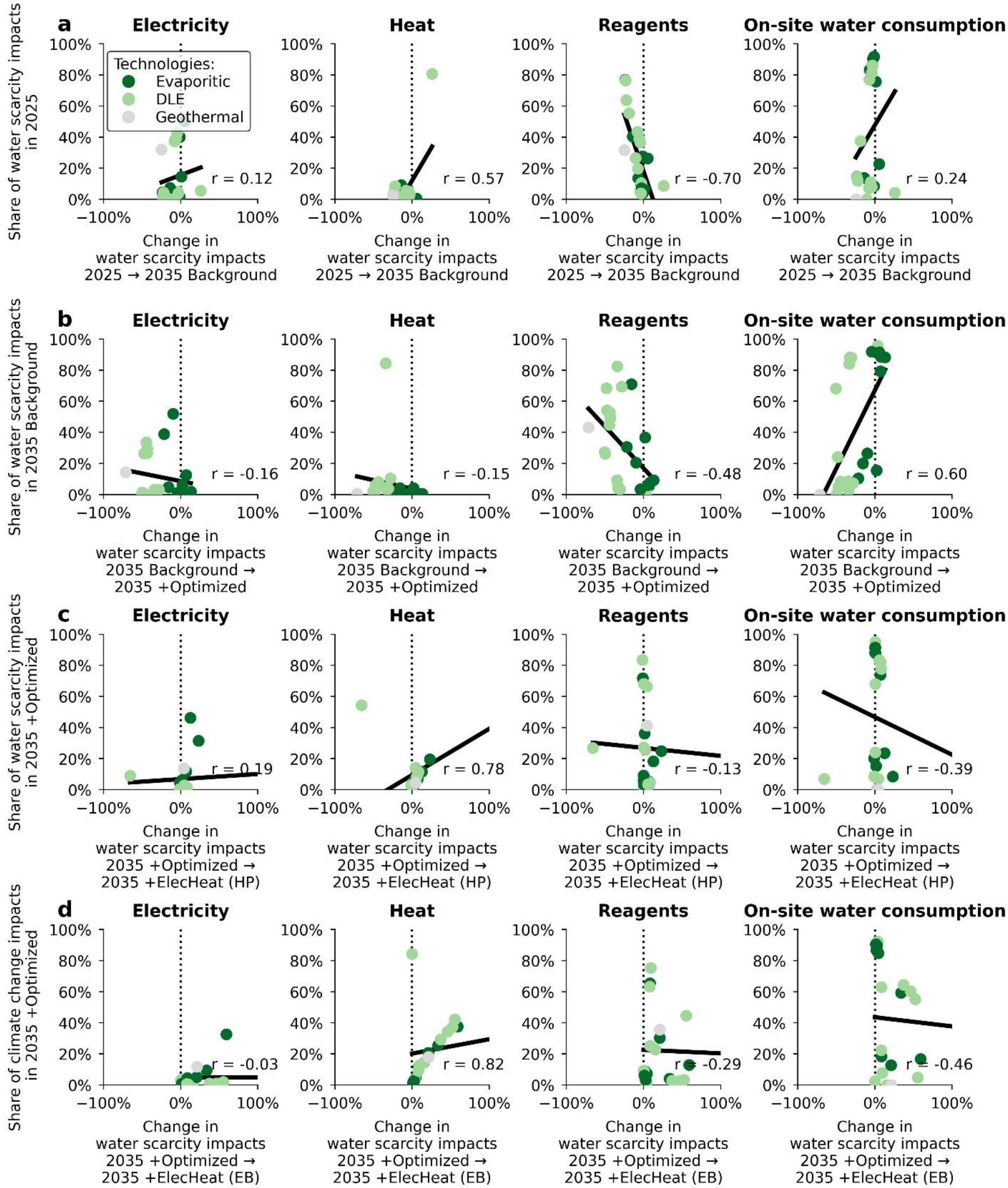

Figure S6. Correlation between the contribution of electricity, heat, reagents, and on-site water consumption to water scarcity impacts and the change in impact across scenarios. The y-axis shows the contribution of each source to climate change impacts in the *2025* (a), *2035 Background* (b) and *2035 +Optimized* (c, d) scenarios. The x-axis shows the relative change in climate change impacts between scenarios: (i) from *2025* to *2035 Background*, (ii) from *2035 Background* to *2035 +Optimized*, (iii) from *2035 +Optimized* to *2035 +ElecHeat* with heat pumps (HP), and (iv) from *2035 +Optimized* to *2035 +ElecHeat* with electric boilers (EB). Negative values indicate impact reduction. Each point represents one project (n = 24)

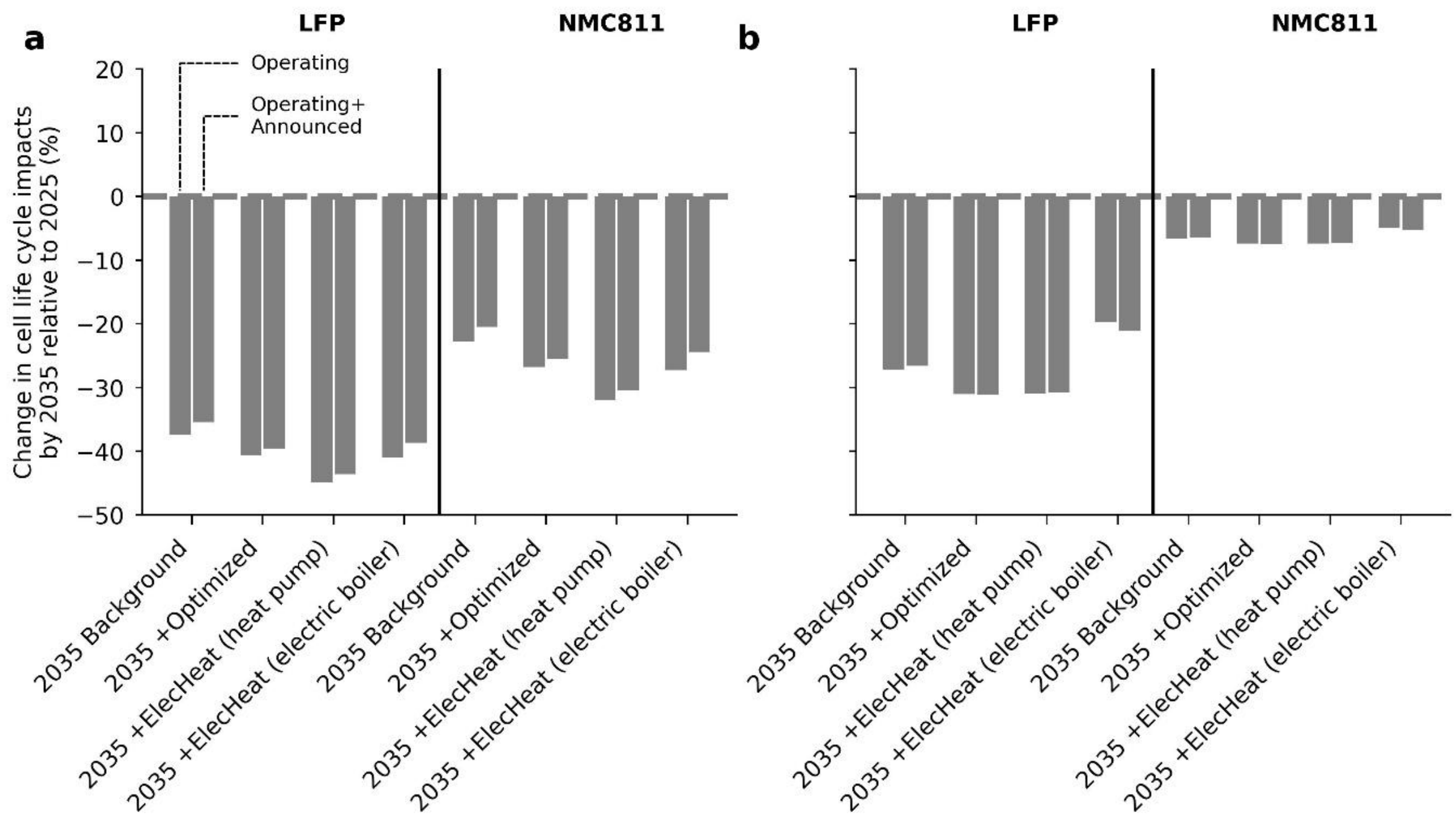

Figure S7. Change in life cycle climate change (a) and water scarcity (b) impacts of LFP and NMC811 battery cell production by 2035 relative to 2025 under alternative Li_2CO_3 supply scenarios.

6. References

(1) S&P Global Market Intelligence. *S&P Capital IQ Pro platform*. https://www.spglobal.com/market-intelligence/en (accessed 2026-04-24).

(2) Buarque Andrade, L.; Frenzel, M.; Bookhagen, B.; Kresse, C.; Schmidt, M.; Nassar, N.; Alonso, E.; Shojaeddini, E.; Sandmann, D. From Exploration to Production: Understanding the Development Dynamics of Lithium Mining Projects. *Resources Policy* **2024**, *99*, 105423. https://doi.org/10.1016/j.resourpol.2024.105423.

(3) Kelly, J. C.; Wang, M.; Dai, Q.; Winjobi, O. Energy, Greenhouse Gas, and Water Life Cycle Analysis of Lithium Carbonate and Lithium Hydroxide Monohydrate from Brine and Ore Resources and Their Use in Lithium Ion Battery Cathodes and Lithium Ion Batteries. *Resources, Conservation and Recycling* **2021**, *174*, 105762. https://doi.org/10.1016/j.resconrec.2021.105762.

(4) Schenker, V.; Pfister, S. Current and Future Impacts of Lithium Carbonate from Brines: A Global Regionalized Life Cycle Assessment Model. *Environ. Sci. Technol.* **2025**, *59* (13). https://doi.org/10.1021/acs.est.4c12619.

(5) Xu, S.; Song, J.; Bi, Q.; Chen, Q.; Zhang, W.-M.; Qian, Z.; Zhang, L.; Xu, S.; Tang, N.; He, T. Extraction of Lithium from Chinese Salt-Lake Brines by Membranes: Design and Practice. *Journal of Membrane Science* **2021**, *635*, 119441. https://doi.org/10.1016/j.memsci.2021.119441.

(6) Li, B. K.; Cheng, H. D.; Ma, H. Z. Boron Isotope Geochemistry of the Lakkor Co Salt Lake (Tibet) and Its Geological Significance. *Geofluids* **2022**, *2022* (1), 3724800. https://doi.org/10.1155/2022/3724800.

(7) Baiyin Zabuye Lithium Industry Co Ltd. Process for Producing Battery-Grade Lithium Carbonate through Processing Carbonate Type Lithium Concentrate by Deep Carbonation Method. CN102502720A.

(8) Ying, J.; Lin, Y.; Zhang, Y.; Yu, J. Developmental Progress of Electrodialysis Technologies and Membrane Materials for Extraction of Lithium from Salt Lake Brines. *ACS EST Water* **2023**, *3* (7), 1720–1739. https://doi.org/10.1021/acsestwater.3c00013.

(9) *Zijin Mining Group Co., Ltd.* https://www.zijinmining.com/ (accessed 2025-04-02).

(10) Schoeneberger, C.; Zhang, J.; McMillan, C.; Dunn, J. B.; Masanet, E. Electrification Potential of U.S. Industrial Boilers and Assessment of the GHG Emissions Impact. *Advances in Applied Energy* **2022**, *5*, 100089. https://doi.org/10.1016/j.adapen.2022.100089.

(11) Šimaitis, J.; Butnar, I.; Sacchi, R.; Lupton, R.; Vagg, C.; Allen, S. Expanding Scenario Diversity in Prospective LCA: Coupling the TIAM-UCL Integrated Assessment Model with Premise and Ecoinvent. *Renewable and Sustainable Energy Reviews* **2025**, *211*, 115298. https://doi.org/10.1016/j.rser.2024.115298.

(12) Laveglia, A.; Sambataro, L.; Ukrainczyk, N.; De Belie, N.; Koenders, E. Hydrated Lime Life-Cycle Assessment: Current and Future Scenarios in Four EU Countries. *Journal of Cleaner Production* **2022**, *369*, 133224. https://doi.org/10.1016/j.jclepro.2022.133224.

(13) Simoni, M.; Wilkes, M. D.; Brown, S.; Provis, J. L.; Kinoshita, H.; Hanein, T. Decarbonising the Lime Industry: State-of-the-Art. *Renewable and Sustainable Energy Reviews* **2022**, *168*, 112765. https://doi.org/10.1016/j.rser.2022.112765.

(14) Laveglia, A.; Ukrainczyk, N.; De Belie, N.; Koenders, E. Decarbonizing the Lime-Based Construction Materials Industry: A Practical Guide for Cradle-to-Gate Life-Cycle Inventory. *Sustainable Production and Consumption* **2023**, *40*, 194–209. https://doi.org/10.1016/j.spc.2023.06.021.

(15) European Lime Association. *A Pathway to Negative CO2 Emissions by 2050 - The Contribution of the Lime Industry to a Carbon-Neutral Europe*; 2023. https://eula.eu/wp-content/uploads/2023/09/WEB_EULA-2030-Climate-Roadmap_Lime-Acts_A4_v19.pdf (accessed 2025-09-20).

(16) European Lime Association. *A Competitive and Efficient Lime Industry: Cornerstone for a Sustainable Europe*; 2014. https://www.eula.eu/wp-content/uploads/2019/02/A-Competitive-and-Efficient-Lime-Industry-Technical-report-by-Ecofys_0.pdf (accessed 2025-09-19).
(17) National Lime Association. *A Roadmap to Carbon Netrality for the U.S. Lime Industry*; 2024. https://www.lime.org/wp-content/uploads/nla_lime_roadmap_final_01_july_24_cc.pdf (accessed 2025-09-23).
(18) Wernet, G.; Bauer, C.; Steubing, B.; Reinhard, J.; Moreno-Ruiz, E.; Weidema, B. The Ecoinvent Database Version 3 (Part I): Overview and Methodology. *Int. J. Life Cycle Assess.* **2016**, *21* (9), 1218–1230. https://doi.org/10.1007/s11367-016-1087-8.
(19) Frauke Schorcht; Ioanna Kourti; Bianca Maria Scalet; Serge Roudier; Luis Delgado Sancho. *Best Available Techniques (BAT) Reference Document for the Production of Cement, Lime and Magnesium Oxide: Industrial Emissions Directive 2010/75/EU (Integrated Pollution Prevention and Control)*; Publications Office: LU, 2013.
(20) Foteinis, S.; Andresen, J.; Campo, F.; Caserini, S.; Renforth, P. Life Cycle Assessment of Ocean Liming for Carbon Dioxide Removal from the Atmosphere. *Journal of Cleaner Production*, 2022, *370*. https://doi.org/10.1016/j.jclepro.2022.133309.
(21) Sacchi, R.; Terlouw, T.; Siala, K.; Dirnaichner, A.; Bauer, C.; Cox, B.; Mutel, C.; Daioglou, V.; Luderer, G. PRospective EnvironMental Impact asSEment (Premise): A Streamlined Approach to Producing Databases for Prospective Life Cycle Assessment Using Integrated Assessment Models. *Renewable and Sustainable Energy Reviews* **2022**, *160*, 112311. https://doi.org/10.1016/j.rser.2022.112311.
(22) Schneider, M. Decarbonisation Pathways for the Australian Lime Sector, 2023.
(23) Mineral Products Association. *MPA Lime - Net Negative 2040 Roadmap*; 2023. https://www.mineralproducts.org/MPA/media/root/Publications/2023/Lime_Net_Negative_2040_Roadmap.pdf (accessed 2025-09-23).
(24) Thieme, C. Sodium Carbonates. In *Ullmann's Encyclopedia of Industrial Chemistry*; Wiley-VCH, Ed.; Wiley, 2000. https://doi.org/10.1002/14356007.a24_299.
(25) USGS. *Mineral Commodity Summaries 2024 - Soda Ash*; 2024.
(26) European Commission. *Integrated Pollution Prevention and Control (IPPC), Reference Document on Best Available Techniques (BREF) in Large Volume Inorganic Chemicals – Solids and Others Industry*; European IPPC Bureau: Brussels, Belgium, 2007. https://bureau-industrial-transformation.jrc.ec.europa.eu/sites/default/files/2019-11/lvic-s_bref_0907.pdf (accessed 2025-09-01).
(27) Lagos, G.; Cifuentes, L.; Peters, D.; Castro, L.; Valdés, J. M. Carbon Footprint and Water Inventory of the Production of Lithium in the *Atacama Salt Flat*, Chile. *Environmental Challenges* **2024**, *16*, 100962. https://doi.org/10.1016/j.envc.2024.100962.
(28) Scott, A. Can Synthetic Soda Ash Survive? *C&EN Global Enterprise*. February 27, 2023, pp 20–22. https://doi.org/10.1021/cen-10107-feature2.
(29) U.S. Environmental Protection Agency. *Technical Support Document for the Soda Ash Manufacturing Sector*; 2009.
(30) Ghaffari, S.; Gutierrez, M. F.; Seidel-Morgenstern, A.; Lorenz, H.; Schulze, P. Sodium Hydroxide-Based CO_2 Direct Air Capture for Soda Ash Production–Fundamentals for Process Engineering. *Ind. Eng. Chem. Res.* **2023**, *62* (19), 7566–7579. https://doi.org/10.1021/acs.iecr.3c00357.
(31) Rumayor, M.; Fernández-González, J.; Domínguez-Ramos, A.; Irabien, A. Deep Decarbonization of the Cement Sector: A Prospective Environmental Assessment of CO2 Recycling to Methanol. *ACS Sustain. Chem. Eng.* **2022**, *10* (1), 267–278. https://doi.org/10.1021/acssuschemeng.1c06118.
(32) We Soda. Ensuring Sustainable Soda Ash Supply, 2022. https://wesoda.com/documents/we-soda-ensuring-sustainable-soda-ash-supply-2022 (accessed 2025-11-11).

(33) Gutierrez, M. F.; Lorenz, H.; Schulze, P. Carbon-Negative Production of Soda Ash: Process Development and Feasibility Evaluation. *Ind. Eng. Chem. Res.* **2025**, *64* (23), 11474–11496. https://doi.org/10.1021/acs.iecr.5c00483.
(34) Rumayor, M.; Dominguez-Ramos, A.; Irabien, A. Toward the Decarbonization of Hard-To-Abate Sectors: A Case Study of the Soda Ash Production. *ACS Sustainable Chem. Eng.* **2020**, *8* (32), 11956–11966. https://doi.org/10.1021/acssuschemeng.0c01598.
(35) USLCI. *National Renewable Energy Laboratory/USLCI - Natural soda ash (Sodium carbonate), at plant*. https://lcacommons.gov/lca-collaboration/National_Renewable_Energy_Laboratory/USLCI_Database_Public/dataset/PROCESS/0d95cc8b-a9a0-3630-a760-1ab4d88257d8?commitId=7d7143983462ce7c48bf8d64bccbe5cafda11c45?gladview=true (accessed 2025-10-13).
(36) Solvay. *How to do more with less (water)*. https://www.solvay.com/en/article/how-do-more-less-water (accessed 2025-11-11).
(37) Aaron Reichl. INDUSTRIAL WATER USE WYOMING'S COLORADO RIVER SYSTEM, 2023. https://csgwest.org/wp-content/uploads/2023/10/Industrial-Water-Challenges-Trona-CGS-West-2023-09-14-Final.pdf (accessed 2025-11-11).
(38) Solvay. *Solvay completes coal phase-out at its US Green River soda ash plant*. Solvay. https://www.solvay.com/en/press-release/solvay-completes-coal-phase-out-its-us-green-river-soda-ash-plant (accessed 2025-10-09).
(39) Solvay. *Solvay Soda Ash takes two giant steps towards carbon neutrality*. https://www.solvay.com/en/article/soda-ash-carbon-neutrality (accessed 2025-10-07).
(40) Krótki, A.; Spietz, T.; Dobras, S.; Chwoła, T.; Tatarczuk, A.; Żórawski, D.; Skowron, K.; Skrzyniecki, D.; Hulisz, P. Carbon Capture Pilot Study in Solvay Soda Ash Process. *Applied Energy* **2025**, *380*, 124995. https://doi.org/10.1016/j.apenergy.2024.124995.
(41) Ewis, D.; Alnouri, S. Y.; El-Naas, M. H. A Techno-Economic Assessment of Conventional and Modified Solvay Processes for CO2 Capture and Reject Brine Desalination. *Journal of Cleaner Production* **2024**, *468*, 143087. https://doi.org/10.1016/j.jclepro.2024.143087.
(42) European Commission. Joint Research Centre. Institute for Prospective Technological Studies. *Best Available Techniques (BAT) Reference Document for the Production of Chlor-Alkali: Industrial Emissions Directive 2010/75/EU (Integrated Pollution Prevention and Control).*; Publications Office: LU, 2014.
(43) Garcia-Herrero, I.; Margallo, M.; Onandía, R.; Aldaco, R.; Irabien, A. Environmental Challenges of the Chlor-Alkali Production: Seeking Answers from a Life Cycle Approach. *Science of The Total Environment* **2017**, *580*, 147–157. https://doi.org/10.1016/j.scitotenv.2016.10.202.
(44) eurochlor. *Chlor Alkali Industry Review 2023-2024*; 2024. https://www.eurochlor.org/wp-content/uploads/2024/08/Chlor_Alkali_Industry_Review_2023_2024.pdf (accessed 2025-09-03).
(45) Scherpbier, E. L. J.; Eerens, H. C. *Decarbonisation Options for the Dutch Chlor Alkali Industry*; 2021.
(46) Li, K.; Fan, Q.; Chuai, H.; Liu, H.; Zhang, S.; Ma, X. Revisiting Chlor-Alkali Electrolyzers: From Materials to Devices. *Trans. Tianjin Univ.* **2021**, *27* (3), 202–216. https://doi.org/10.1007/s12209-021-00285-9.
(47) US EPA. *Hydrochloric Acid Supply Chain Profile*; 2023.
(48) Vera, M. L.; Torres, W. R.; Galli, C. I.; Chagnes, A.; Flexer, V. Environmental Impact of Direct Lithium Extraction from Brines. *Nat Rev Earth Environ* **2023**, *4* (3), 149–165. https://doi.org/10.1038/s43017-022-00387-5.
(49) Boulay, A.-M.; Bare, J.; Benini, L.; Berger, M.; Lathuillière, M. J.; Manzardo, A.; Margni, M.; Motoshita, M.; Núñez, M.; Pastor, A. V.; Ridoutt, B.; Oki, T.; Worbe, S.; Pfister, S. The WULCA Consensus Characterization Model for Water Scarcity Footprints: Assessing Impacts of Water Consumption

Based on Available Water Remaining (AWARE). *Int J Life Cycle Assess* **2018**, *23* (2), 368–378. https://doi.org/10.1007/s11367-017-1333-8.
(50) Flexer, V.; Baspineiro, C. F.; Galli, C. I. Lithium Recovery from Brines: A Vital Raw Material for Green Energies with a Potential Environmental Impact in Its Mining and Processing. *Science of The Total Environment* **2018**, *639*, 1188–1204. https://doi.org/10.1016/j.scitotenv.2018.05.223.
(51) Schomberg, A. C.; Bringezu, S.; Flörke, M. Extended Life Cycle Assessment Reveals the Spatially-Explicit Water Scarcity Footprint of a Lithium-Ion Battery Storage. *Commun Earth Environ* **2021**, *2* (1), 1–10. https://doi.org/10.1038/s43247-020-00080-9.
(52) Chordia, M.; Wickerts, S.; Nordelöf, A.; Arvidsson, R. Life Cycle Environmental Impacts of Current and Future Battery-Grade Lithium Supply from Brine and Spodumene. *Resources, Conservation and Recycling* **2022**, *187*, 106634. https://doi.org/10.1016/j.resconrec.2022.106634.
(53) Mas-Fons, A.; Horta Arduin, R.; Loubet, P.; Pereira, T.; Mahmud Parvez, A.; Sonnemann, G. Carbon and Water Footprint of Battery-Grade Lithium from Brine and Spodumene: A Simulation-Based LCA. *Journal of Cleaner Production* **2024**, 142108. https://doi.org/10.1016/j.jclepro.2024.142108.
(54) Schenker, V.; Oberschelp, C.; Pfister, S. Regionalized Life Cycle Assessment of Present and Future Lithium Production for Li-Ion Batteries. *Resources, Conservation and Recycling* **2022**, *187*, 106611. https://doi.org/10.1016/j.resconrec.2022.106611.
(55) Peñaloza-Pacheco, L.; Triantafyllou, V.; Martínez, G. The Non-Green Effects of *Going Green*: Local Environmental and Economic Consequences of Lithium Extraction in Chile. *Journal of Environmental Economics and Management* **2026**, *136*, 103257. https://doi.org/10.1016/j.jeem.2025.103257.
(56) Du, W.; Chen, L.; He, Y.; Wang, Q.; Gao, P.; Li, Q. Spatial and Temporal Distribution of Groundwater in Open-Pit Coal Mining: A Case Study from Baorixile Coal Mine, Hailaer Basin, China. *Geofluids* **2022**, *2022* (1), 8753217. https://doi.org/10.1155/2022/8753217.
(57) Park, E.; Hackney, C. R.; Tran, D. D.; Bendixen, M.; Best, J.; Yuen, K. W.; Runeckles, H.; Chyon, M. S. A.; Kästner, K.; Kumar, S.; Yusuf, H.; Lamb, V.; Iversen, L. L.; Spiller, A.; Sinha, R.; Silva, E. F. da; Alcantara, E.; Wu, C.; Lu, C.; Jian, C.; Wang, J.; Thien, N. D.; Feng, L.; Patel, P. P.; Huang, J.; Switzer, A. D. River Sand and Gravel Mining: Global Drivers, Impacts, and Pathways for Sustainable Management. *Reviews of Geophysics* **2026**, *64* (3), e2024RG000868. https://doi.org/10.1029/2024RG000868.
(58) Halkes, R. T.; Hughes, A.; Wall, F.; Petavratzi, E.; Pell, R.; Lindsay, J. J. Life Cycle Assessment and Water Use Impacts of Lithium Production from Salar Deposits: Challenges and Opportunities. *Resources, Conservation and Recycling* **2024**, *207*, 107554. https://doi.org/10.1016/j.resconrec.2024.107554.
